# Nature’s Anaerobic Toolkit: Glycyl Radical Enzymes and Their Expanding Functional and Mechanistic Diversity

**DOI:** 10.1021/acs.biochem.6c00318

**Published:** 2026-06-26

**Authors:** Christa N. Imrich, Ankush Chakraborty, Lindsey R. F. Backman, Mary C. Andorfer, Catherine L. Drennan

**Affiliations:** † Department of Chemistry, 2167 Massachusetts Institute of Technology, Cambridge, Massachusetts 02139, United States; ‡ Department of Chemistry, 3078 Michigan State University, East Lansing, Michigan 48824, United States; § 2187 The Whitehead Institute for Biomedical Research, Cambridge, Massachusetts 02142, United States; ∥ Department of Biology, 2167 Massachusetts Institute of Technology, Cambridge, Massachusetts 02139, United States; ⊥ HHMI, 2167 Massachusetts Institute of Technology, Cambridge, Massachusetts 02139, United States

**Keywords:** glycyl radical, thiyl radical, radical S-adenosyl-L-methionine, C–C bond formation, lyase, eliminase, decarboxylase, bacterial microcompartments

## Abstract

Nature has devised an array of enzymatic cofactors that enable challenging chemical transformations to occur on a time scale compatible with biological life. The glycyl radical enzyme (GRE) superfamily, for example, highlights the catalytic power of protein-based amino acid radicals. GREs utilize a persistent radical housed on the α-carbon of a C-terminally conserved glycine residue to accomplish diverse chemical reactions in anaerobic environments. This radical cofactor is post-translationally installed on the mature, folded protein by a radical *S*-adenosyl-L-methionine (RS) activating enzyme (GRE-AE; activase) specific to each GRE. The catalytic repertoire of GREs is diverse, spanning ribonucleotide reduction for DNA synthesis, acetyl-CoA formation in anaerobic primary metabolism, dehydration reactions in secondary metabolism, decarboxylation reactions in aromatic amino acid fermentation, and hydrocarbon activation in anaerobic bacteria, enabling their survival on crude oil as a carbon source. Pyruvate formate lyase (PFL), the founding member of the GRE superfamily, was first identified several decades ago. In recent years, sequencing technology has enabled the annotation of thousands of unique GRE-encoding sequences. Several dozen of the identified GREs have been characterized further, providing insight into the structure, mechanism, and regulation of this multitudinous and diverse enzyme family. Here, we introduce the superfamily, summarize what is known about their activation and catalysis, note conserved structural features, expand on the previously identified GRE classes, highlight GRE-associated bacterial microcompartments, and discuss the future and outlook of the GRE superfamily.

## Graphical abstract

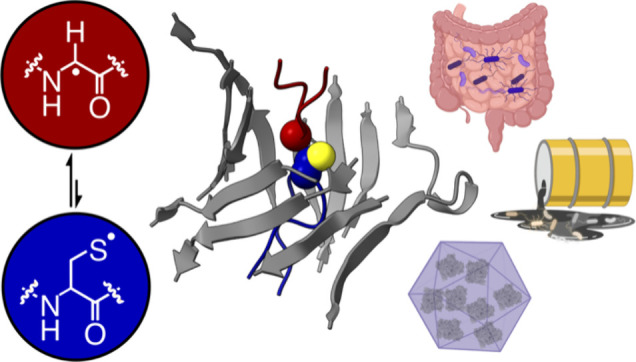

## 1 Introduction to Glycyl Radical Enzymes

Glycyl radical enzymes (GREs) are a large superfamily of strictly anaerobic enzymes that use a protein-based glycyl radical cofactor to perform catalysis. GREs are typically 80–100 kDa in size, consist of a 10-stranded β/α-barrel architecture, and are canonically thought to function as homodimers, as demonstrated by the GRE pyruvate formate lyase (PFL) (Figure 1A,B).^1–3^ Within the GRE polypeptide, a conserved C-terminal domain referred to as the glycyl radical domain (GRD) contains the conserved fingerprint motif RVx**G**[FWY]­x_6–8_[FL]­x_4_Qx_2_[IV]­x_2_R^4^ and contains the glycine residue that is converted into the glycyl radical cofactor (Figure 1A,B). In addition to the conserved glycine, there is also a conserved cysteine residue that is essential for catalysis. Two finger loops house these catalytically essential glycine and cysteine residues and are therefore referred to as the “Gly loop” and “Cys loop”.^5–10^ The Gly and Cys loops meet in the center of the β-barrel and comprise part of the buried active site (Figure 1B). In PFL, there are two conserved cysteines on the Cys loop; however, the majority of GREs have a single cysteine (Figure 1A). The glycyl radical is post-translationally installed by GRE activating enzymes (GRE-AEs), all of which are members of the radical *S-*adenosyl-L-methionine (RS) superfamily of enzymes.^2,11–13^ As such, GRE-AEs use a catalytic [4Fe-4S]^2+/1+^ cluster^14^ to homolytically cleave a molecule of *S-*adenosyl-L-methionine (AdoMet), which forms a highly reactive 5′dAdo radical species (5′dAdo•) (Figure 1C).^15–17^ The 5′dAdo• then stereoselectively abstracts the pro-*S* hydrogen atom from the glycine residue, generating the glycyl radical at the α-carbon (Figure 1D).^18^ The conserved glycine residue in the folded protein is buried ∼20 Å beneath the surface of the enzyme. Consequently, there must be a dramatic conformational change in the enzyme, where the GRD flips out of the active site, to allow for post-translational installation of the glycyl radical (Figure 1E). In the catalytic cycle of GREs, the glycyl radical first abstracts a hydrogen atom from a nearby, conserved cysteine thiol, forming a transient thiyl radical (Figure 1D).^19^ The thiyl radical initiates catalysis, typically through H-atom abstraction from substrate (Figure 1D). The resultant substrate radical rearranges to form a product radical, which reabstracts a hydrogen atom from the cysteine thiol to regenerate the thiyl radical. The thiyl radical then reforms the glycyl radical for storage in between rounds of turnover (Figure 1D). Herein, we discuss GRE activation by their activating enzymes, radical cofactors in GREs, GRE structures, GRE classifications, mechanisms of catalysis employed by GREs, and GRE associated bacterial microcompartments. As the first step in GRE function, we begin by discussing GRE activation and the enzymes responsible for that activation.

**Figure 1:**
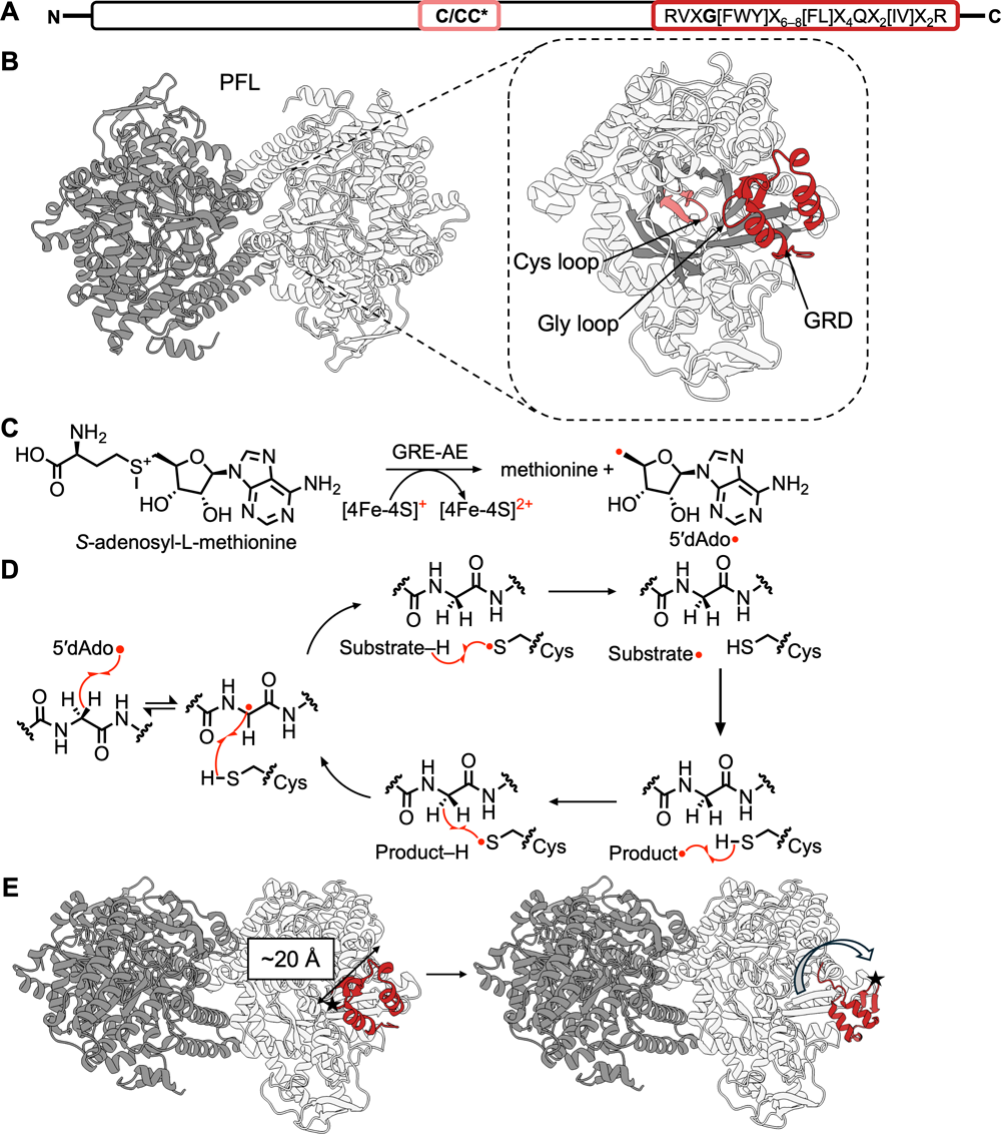
Introduction to glycyl radical enzymes. (A) Domain map of PFL showing the GRD (red) and conserved cysteines on the Cys loop (pink). *Only PFLs and TdcEs are known to have a two-cysteine motif on the Cys loop. (B) Pyruvate formate lyase structure (PDB ID: 1H18) showing the GRE homodimer. Inset shows 10-stranded β-barrel, Gly, and Cys loops. (C) Reaction scheme of AdoMet cleavage by GRE-AEs. (D) Conserved catalytic mechanism of GREs. (E) The glycine that houses the radical cofactor is buried in the GRD (red) and must flip out of the active site for post-translational radical installation by the AE. Site of glycyl radical is indicated with a black star.

## 2 GRE Activation and Activating Enzymes

### 2.1 Overview of GRE-AE Structure and Function

GRE-AEs are members of the RS superfamily of enzymes and, as such, contain a canonical RS domain comprised of a 6-stranded parallel β-sheet (Figure 2A).^20^ The vast majority of identified GRE-AEs contain the RS domain and an auxiliary ferredoxin-like domain^13,21^ with eight additional conserved cysteine residues that could ligate up to two additional [4Fe-4S] clusters for a total of three [4Fe-4S] clusters. Notably, PFL-AE and class III ribonucleotide reductase (RNR)-AE solely contain the RS domain. The structure and function of the auxiliary ferredoxin domain is not known, though it has been suggested to be required for the generation of a long-lasting glycyl radical in the hydroxyphenylacetate decarboxylase (HPAD) system.^22^ GRE-AE function can be broken into three distinct overall steps: (1) reduction of the active site [4Fe-4S] cluster; (2) homolytic cleavage of AdoMet to form 5′dAdo•; and (3) abstraction of a hydrogen atom from the conserved C-terminal glycine residue in the substrate GRE. The structure of *E. coli* PFL-AE is the only experimental structure available of an activase (Figure 2A).^20^ Two PFL-AE structures were solved by Vey et al.: one of PFL-AE with a partially disordered AdoMet bound, and one of PFL-AE with an ordered AdoMet and a 7-mer peptide mimic of the PFL Gly loop bound in the active site of the AE. More recently, additional structures of PFL-AE were determined with the goal of improving crystallization of the enzyme.^23^ Taken together, these structures and decades of biochemical work have informed our understanding of GRE-AEs. Below, we briefly summarize what is known about each of the three aforementioned overall steps of GRE-AE function. We recommend a recent review by Broderick and co-workers for those seeking additional information on radical generation by RS enzymes.^24^

**Figure 2:**
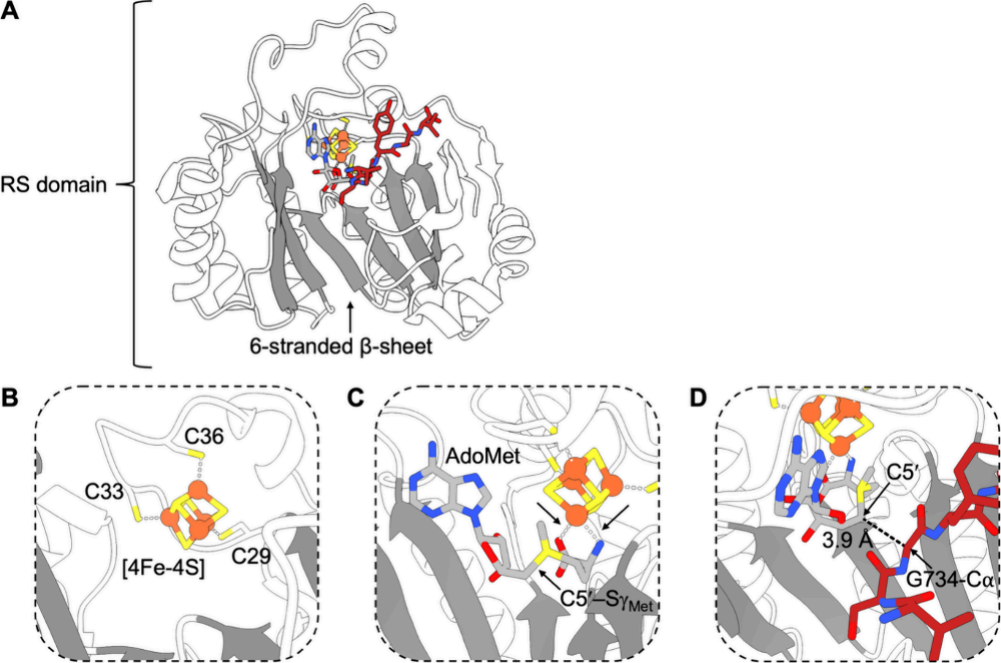
X-ray crystal structure of PFL-AE. (A) The overall structure of PFL-AE from *E. coli* shows an RS enzyme fold with a [4Fe-4S] cluster, AdoMet, and the 7-mer peptide bound (PDB ID: 3CB8). The 7-mer peptide is shown in red. (B) The [4Fe-4S] cluster is coordinated by C29, C33, and C36. (C) AdoMet coordinates the [4Fe-4S] cluster via its amino and carboxylate moieties, indicated by black arrows. The 5′C–Sγ_Met_ bond which is homolytically cleaved is indicated with an arrow and labeled. (D) A 7-mer peptide mimicking the glycine loop of PFL (red) is bound in the active site of PFL-AE, with a Gly–Cα to AdoMet–C5′ distance consistent with other RS enzymes. All Fe atoms are shown in orange. All S atoms are shown in yellow.

### 2.2 Fe-S Cluster Reduction

To homolytically cleave AdoMet, the [4Fe-4S] cluster in PFL-AE must first be assembled and reduced to the 1+ oxidation state. Fe-S cluster biosynthesis is outside the scope of this review and is reviewed elsewhere.^25^ Consistent with all RS enzymes, GRE-AEs contain a conserved CX_3_CX_2_C motif that ligates the catalytic [4Fe-4S] cluster (Figure 2B). In the crystal structure of PFL-AE, these cysteines are Cys29, Cys33, and Cys36 (Figure 2B).^20^ Natively expressed *E. coli* PFL-AE is known to use flavodoxin as an electron source in vivo to reduce the [4Fe-4S] cluster from the 2+ to the 1+ oxidation state.^26^ Small molecule reductants, including photoreductants such as 5-deazariboflavin, have also long been used as electron donors to reduce the [4Fe-4S] cluster of recombinantly expressed PFL-AE.^27^ Under single-turnover conditions (no external reductant to rereduce the cluster), PFL-AE and class III RNR-AE generate stoichiometric amounts of glycyl radical, as shown by EPR spin quantitation.^14,28^ The glycyl radical is generated via the intermediate 5′-deoxyadenosyl radical (5′dAdo•), as discussed below.

### 2.3 AdoMet Cleavage and Formation of 5′dAdo•

AdoMet coordinates the unique Fe of the [4Fe-4S] cluster via its amino and carboxylate moieties, which has been confirmed spectroscopically^29,30^ and structurally^20^ (Figure 2C). A structure of PFL-AE with an ordered AdoMet molecule was only captured when the 7-mer peptide mimicking the Gly loop was also bound to the enzyme. When the peptide was absent, AdoMet was partially disordered.^20^ GRE-AEs, like most other RS enzymes, homolytically cleave the 5′C–Sγ_Met_ bond to form the 5′dAdo• that acts on substrate. Despite its involvement in thousands of RS enzymes, the 5′dAdo•, due to its instability, was not directly spectroscopically captured until recently.^17^ GRE-AEs have been shown to cleave AdoMet uncoupled from glycyl radical installation, but this nonproductive cleavage is significantly reduced without the partner GRE.^31–34^ Once the 5′dAdo• has been formed, the final step in GRE activation is the installation of the glycyl radical cofactor.

### 2.4 Installation of the Glycyl Radical

The 5′dAdo• is a potent oxidant, and formation of the glycyl radical is thermodynamically downhill due to the difference in bond dissociation energies between the C5′–H of deoxyadenosine and the Cα–H of glycine.^35^ Hydrogen atom abstraction from the conserved C-terminal glycine of the GRE polypeptide by 5′dAdo• is direct and stereospecific.^18^ In vitro, GRE activation is rarely perfectly efficient. For example, PFL has been reported to reach only about 0.5 radicals per polypeptide.^36^ Similar partial loading has been observed for hydroxyproline dehydratase (HypD) (about 0.5 radicals per polypeptide).^37^ It remains an open question whether this partial loading reflects half-site reactivity. Other GREs show even less activation in vitro, such as benzylsuccinate synthase (BSS) and HPAD (∼0.25 radicals per monomer) or the newly characterized C–O lyases YbiW and PflD (0.02 and 0.06 radicals per monomer, respectively).^32,38,39^ The presence of GRE-AE is suggested to impact the conformational equilibrium of GREs. In one study, the PFL-AE:PFL ratio strongly affected both the rate of radical installation and its persistence. At lower PFL-AE:PFL ratios (1:10 or 1:100), glycyl radical installation was slower, but the radical persisted longer once formed.^36^ In contrast, at a 1:1 ratio, radical installation was much faster, but more rapid radical loss was observed. The structure of PFL-AE with the 7-mer peptide mimicking the Gly loop provides the only structural insight to date on GRE activation and shows an AdoMet–C5′ to Gly734–Cα distance of 3.9 Å (Figure 2D).^20^ This distance is consistent with that seen in other RS enzyme structures between the AdoMet–C5′ and the respective substrates.^40^ After glycyl radical generation in the AE active site, the GRD must flip back into the buried active site of the GRE for catalysis to occur; however, the mechanism of this step is not well understood.

### 2.5 GRE-AE Specificity

Specificity of GRE-AEs for their cognate GREs has not been studied in-depth; however, a few insightful studies have revealed factors that regulate GRE:GRE-AE specificity. One study demonstrated that PFL-AE from *E. coli* could install glycyl radical on a 7-mer peptide mimicking the Gly loop of PFL (RVS**G**YAV), but not on a 7-mer peptide mimicking the Gly loop of class III RNR (RVC**G**YLG).^18^ PFL-AE is also unable to install glycyl radical on the full-length BSS polypeptide in vitro.^41^ These results suggest that GRE-AE specificity for its partner GRE is dependent upon Gly loop sequence. There are few examples of cross-reactivity of GRE-AEs for non-native GRE counterparts, and they are often between highly similar GREs or homologues of the same GRE. For example, HPAD-AE from *Clostridioides difficile* or *C. scatologenes* could activate either *Cd*HPAD or *Cs*HPAD.^38^ These HPADs have identical Gly loop sequences (RVA**G**FTQ). Additionally, isopropylbenzylsuccinate synthase (IBSS)-AE from *Thauera* sp. pCyN2 could activate both its native IBSS and BSS from *Thauera aromatica*.^32^ The Gly loops of BSS and IBSS from these species are conserved, differing only in the identity of the aromatic residue immediately following the essential Gly (RVS**G**[Y/F]­SA). Finally, the PFL-AE from *E. coli* has also been shown to activate the 2-ketobutyrate formate lyase (TdcE) from *E. coli*.^42^ Again, the Gly loop sequence between these two enzymes is conserved (RVS**G**YAV). The full extent to which Gly loop sequence determines specificity has not been determined. A 7-mer peptide mimicking the PFL Gly loop with a tyrosine to phenylalanine substitution (RVSG­[**Y→F**]­AV) is still activated by PFL-AE.^18^ Additional studies to probe the ability of 7-mer peptides to be activated by various GRE-AEs would surely illuminate additional details of this regulation. Moreover, Gly loop identity is unlikely to be the only determinant of partner recognition; other features likely contribute to specificity but remain poorly defined.

## 3 Radical Cofactors in GREs

Nature’s choice of glycine as the radical-storing residue in GREs has long been contemplated.^43^ The glycyl radical is thought to be stabilized by the captodative effect, in which the presence of an electron donating group and an electron withdrawing group flanking the radical species enables resonance-based stabilization of the radical.^44^ Computational studies suggest that Cα radical stability reflects a balance between substituent stabilization and steric destabilization imposed by the planar radical geometry. For example, side-chain substituents can stabilize the radical through hyperconjugation, but nonbonding repulsion with the backbone can offset this effect, leading to Cα–H bond strengths that are often similar to that of glycine.^44^ Other studies argue that the steric penalty dominates for many residues, making substituted Cα radicals less stable than glycyl radicals.^45,46^ Moreover, radical stability can be strongly conformation-dependent because different rotamers and backbone geometries modulate both steric clash and orbital alignment.^46^ The emerging discovery of other aminoacyl radical enzymes (AAREs)^47^ is consistent with this view: even if glycine is often a favorable radical reservoir, alanine, serine, and threonine can also support radical storage in the right structural context. To date, all structures of GREs have been in the inactivated state (glycine); structural characterization of an activated GRE or AARE will no doubt uncover more about how local structure and dynamics tune radical stability, and why different enzymes can use different amino acids as radical reservoirs.

The glycyl radical cofactor is readily detectable by electron paramagnetic resonance (EPR) spectroscopy due to its S=12 spin state and shows a characteristic doublet signal with *g* = ∼2, which is completely abolished by exposure of the protein to oxygen.^11,48,49^ The radical is centered on the glycine Cα, and the doublet splitting observed in EPR spectra arises from hyperfine coupling to the α-proton.^50^ GREs are ancient, predating the Great Oxygenation Event, and exhibit severe oxygen intolerance. In vitro under anaerobic conditions, the glycyl radical is remarkably stable, with an estimated half-life of at least 24 h in PFL,^49,51,52^ and persistence for up to 11 days in BSS.^53^ However, upon exposure to oxygen, GREs are rapidly inactivated.^11^ The oxygen-dependent inactivation of GREs occurs via peroxyl and sulfinyl radical formation, ultimately resulting in the cleavage of the polypeptide backbone.^54,55^ Due to this oxygen-sensitivity, GREs are found in microbes that survive in anaerobic environments, such as the human gut.

Most mechanistic models invoke rapid interconversion between the glycyl radical and a thiyl radical to enable radical transfer during catalysis (see Figure 1D). In thermodynamic terms, the position of this equilibrium depends on the relative energetics of the two states, often framed in terms of the Gly Cα–H versus Cys Sγ–H bond strengths and the corresponding stabilities of the glycyl and thiyl radicals in the protein environment. Across the literature, reported energetics for Gly Cα–H versus Cys Sγ–H span a wide range, largely because different studies compute different quantities (intrinsic X–H BDEs, radical stabilization energies, or radical transfer thermodynamics) using models and methods with different sensitivity to local structure and environment. Using isodesmic DFT models, several groups have reported BDEs of ∼78–83 kcal/mol for Gly Cα–H and ∼83–89 kcal/mol for the Cys Sγ–H.^44,56–61^ These ranges likely reflect differences in model construction, basis set, functional, and other computational details used to mimic the surrounding protein environment.

Consistent with the long-standing view that the glycyl radical is the dominant resting state, it is the only species directly observed by EPR spectroscopy, whereas the thiyl radical has remained spectroscopically elusive. This observation suggests that the glycyl radical is lower in energy under experimentally accessible conditions, although it does not by itself establish the thermodynamics of radical exchange. If the glycyl radical is substantially more stable than the thiyl radical, then formation of the thiyl intermediate would be thermodynamically uphill and could slow catalysis. Thermodynamic calculations from the Zipse group suggest that protein-bound glycyl and thiyl radicals have comparable stabilities, making glycyl-thiyl radical exchange effectively thermoneutral.^62^ Recent multiscale modeling of BSS further emphasizes that glycyl-thiyl radical transfer depends on more than static, isolated X–H bond strengths.^63^ In this study, the forward and reverse H-transfer steps are predicted to be principally reversible, with rates strongly shaped by active-site geometry and substrate/product binding.

Despite the lack of direct spectroscopic observation, the existence of a transient thiyl radical is confirmed by indirect biochemical markers and kinetic data. For example, the characteristic glycyl radical doublet observed by EPR spectroscopy has been shown to collapse to an apparent singlet upon incubation of the activated protein in D_2_O for certain systems. This collapse of the doublet was interpreted as deuterium incorporation into Gly through hydrogen atom transfer (HAT) with Cys.^19,48,49,53,64–66^ This observation suggests that the HAT is not strictly stereospecific. Recent modeling of BSS supports this interpretation by showing that abstraction from the alternate Gly Cα–H is possible, although it is predicted to occur more slowly.^63^ Since Cys is not required for the initial formation of the glycyl radical, site-directed mutagenesis can be used to evaluate how specific mutations influence the radical equilibrium. Mutation of the Cys to Ala or Ser abolishes the exchange, further supporting that the apparent singlet is caused by HAT between Gly and Cys.^19,55^ Recently, trapping of the thiyl radical in PFL with methacrylate was reported by Caceres et al.^67^ When methacrylate is used as a substrate analog of pyruvate, it irreversibly covalently attaches to C418–Sγ, generating a C2 tertiary alkoxyradical captured by EPR spectroscopy and resolved by DFT calculations. Not all GREs under all conditions show this deuterium exchange/collapse to an apparent singlet. PFL^19,49,64^ and BSS^48,53,65,66^ do under certain conditions. RNR does not show exchange.^68,69^ A lack of exchange can be attributed to two different phenomena - true stereospecificity of HAT between Gly and Cys or a lack of thiyl radical formation.

## 4 GRE Structure

### 4.1 The Overall Architecture of GREs

GREs are globular proteins that, as mentioned above, consist of a core 10-stranded β-barrel made up of two antiparallel 5-stranded β-sheets which are surrounded by α-helices (Figure 3A,B). Additionally, GREs all contain two β-hairpin motifs that house the catalytically essential glycine and cysteine residues. As mentioned in Section 1, these motifs are termed the Gly loop and Cys loop, respectively, and meet in the center of the barrel to form part of the buried active site (Figure 3A,B). A buried active site enables the precise positioning of substrate and highly reactive intermediates to allow for a specific chemical transformation to occur while mitigating off-pathway reactions. At the N-terminus of the polypeptide are helices that form a conserved dimer interface, which is discussed in-depth in section 4.4 below. Sequentially, the first half-barrel precedes the Cys loop and then is followed by the second half barrel (Figure 3A,B). At the C-terminus of the polypeptide, a helix−β-hairpin–helix motif comprises the glycyl radical domain (GRD), which houses the Gly loop (Figure 3C). The GRD is located on the periphery of the symmetrical GRE homodimer, which positions it well to flip out of the active site for glycyl radical installation by a GRE-AE (Figure 1E). Between β2 and β3 of the barrel is a short, structurally conserved “cap” helix that is positioned to cover one end of the β-barrel (Figure 3C). All GREs retain the above structural features within the catalytic subunit.

**Figure 3:**
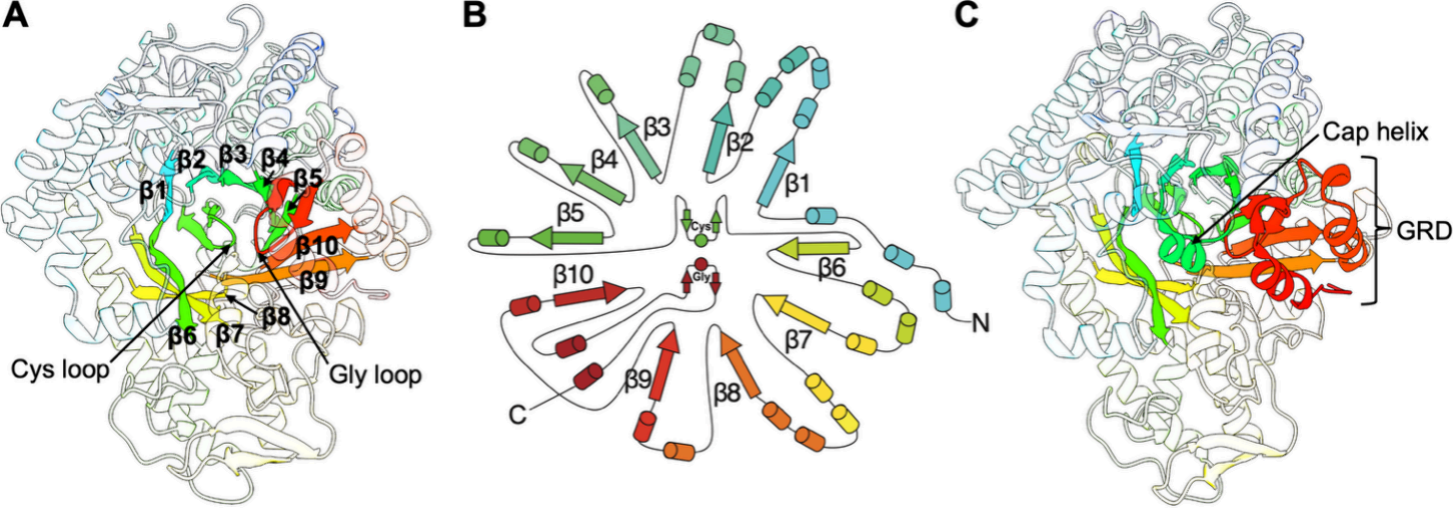
Overall architecture of GREs. (A) GREs are made of two antiparallel 5-stranded β-sheets. The first half β-sheet is followed by the Cys loop, the second half β-sheet, and then the Gly loop. The Cys and Gly loops meet in the center of the barrel to form part of the buried active site. Structural architecture demonstrated with PFL (PDB ID: 1H18). (B) Topology diagram of GREs showing the conserved 10-stranded β/α-barrel. (C) The GRD is located at the C-terminus of the GRE at the periphery of the structure such that it can be flexible for post-translational radical installation. The cap helix is conserved across GREs, located between β2 and β3, and covers one end of the barrel.

### 4.2 GRE Small Subunit Structure and Function

In addition to the larger catalytic subunit described in Section 4.1, some GREs form complexes with additional smaller subunits. In these systems, the catalytic subunit is referred to as the α subunit. Currently, the presence of small subunits has been observed in three GREs: HPAD,^70^ BSS,^71^ and 1-methylalkylsuccinate synthase (MASS).^72–74^ HPAD contains only one small subunit, γ;^70,75^ BSS contains two, β and γ;^5,71,76^ and MASS contains three, β, γ, and δ (Figure 4A–C).^74,77,78^ The small subunits HPADγ, BSSβ, BSSγ, MASSβ, and MASSγ structurally resemble high-potential [4Fe–4S] cluster proteins (HiPIPs)^79^ (Figure 4D). MASSδ, in contrast, is the sole accessory GRE subunit that structurally resembles a rubredoxin protein rather than a HiPIP protein (Figure 4E).^77,80^ The redox potential for the 3+/2+ redox couple of the BSS [4Fe–4S] clusters has been measured by protein film voltammetry at + 76 mV,^5^ on the low end of the range for HiPIP proteins (+100 to +350 mV).^81^ A redox potential for the 2+/1+ redox potential has been estimated as being below −450 mV by redox titrations.^81,82^ The γ subunit in each GRE is bound ∼30 Å away from the GRD and active site, and closer to the dimerization interface (Figure 4A–C). The γ subunits of each multisubunit GRE cover a large hydrophobic patch of the α subunit and are hypothesized to primarily play a role in enzyme solubility.^70,76,77^ Recombinant expression of the BSS and MASS α subunits alone result in protein found entirely in inclusion bodies.^5,76,77^ The HPAD α subunit can be expressed and purified as a homodimer (α_2_) that is almost entirely catalytically inactive.^70,83^ A stable heterooctamer (α_4_γ_4_) is formed when HPAD’s α and γ subunits are recombinantly coexpressed and achieves robust product formation.^83^ Phosphorylation of the HPAD γ subunit was identified and suggested to play a role in oligomerization.^70^ However, phosphorylation of the HPAD α subunit has also been demonstrated, and the exact role of these phosphorylation events is unclear.^38^

**Figure 4:**
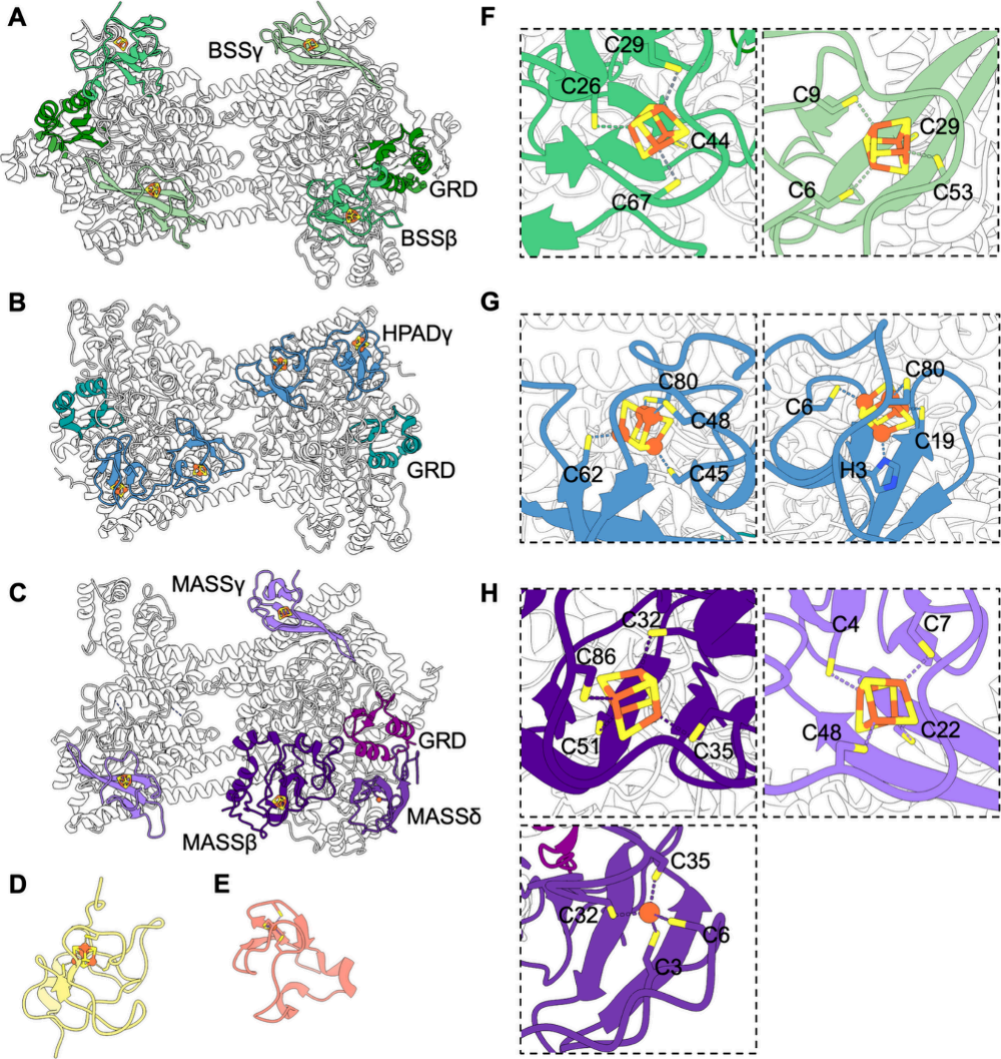
GRE small subunits. (A) Overall BSS architecture (PDB ID: 9PZJ) showing β (dark sea green), γ (sea green), and GRD (green). (B) Overall architecture of the HPAD heterotetramer (α_2_γ_2_) (PDB ID = 2YAJ) showing γ (steel blue) and GRD (dark cyan). (C) Overall MASS architecture (PDB ID: 9O8U) showing β (indigo), γ (medium purple), δ (rebecca purple), and GRD (purple). (D) HiPIP protein (PDB ID: 1ISU). (E) Rubredoxin protein (PDB ID: 6RXN). (F) BSS β and γ subunits harbor [4Fe-4S] clusters. (G) HPAD γ subunit harbors two [4Fe-4S] clusters, one ligated by four Cys, and one ligated by three Cys and one His. (H) MASS β and γ each harbor a [4Fe-4S] cluster, whereas MASS δ harbors a mononuclear nonheme Fe cofactor. All Fe atoms are colored orange. All S atoms are colored yellow.

The β subunit of BSS is bound such that its C-terminus contacts the GRD. In addition, a loop of BSSβ covers the substrate channel, which is discussed in detail in Section 4.3 (Figure 4A).^5,84^ Funk et al. also showed that BSSβ regulates the outward movement of the glycyl radical domain; that in the absence of BSSβ, the GRD has moved out of the active site such that the glycyl radical loop is slightly farther away from the Cys loop by 0.6 Å.^5^ Without BSSβ present in the crystal structure, the B-factors for the GRD are higher, further indicating a role for BSSβ in restricting GRD movement. More recent work revealed the connection between GRD movement and glycyl radical installation, showing that activation occurs slower in the presence of BSSβ.^32^ However, this work also showed that once the enzyme is activated, i.e., the glycyl radical is installed, BSSβ is necessary for turnover of the enzyme to produce product.^32^ Additionally, Anas and co-workers in 2025 showed that binding of BSSβ triggers thiyl radical formation in activated BSS even in the absence of substrate,^53^ suggesting that BSSβ must alter the positioning of the GRD and Gly loop in some way that promotes radical transfer to the catalytic Cys. In the absence of BSSβ, this transfer is not promoted, and the glycyl radical in BSS persists for up to 11 days.^53^ The persistence of this radical is severely shortened in vitro when BSSβ is present. Altogether, BSSβ appears to regulate glycyl radical installation and thiyl radical formation in BSS, presumably by modulating the GRD and its Gly loop positioning in both cases.

In MASS, the β and δ subunits occupy the same general region as the β subunit does in BSS, but together they span a larger area on the surface of the α subunit (Figure 4C).^77^ MASSβ contains a loop that binds over the predicted substrate channel, likely regulating hydrocarbon entry similar to BSSβ. Unlike BSSβ, MASSβ is larger and is rotated ∼90° as compared with BSSβ. It contains an extra N-terminal helix that has also been proposed to regulate entry to the substrate channel. This additional gating may be particularly important for MASS because *n*-alkane substrates are more conformationally flexible than toluene and may therefore require tighter β-mediated control of α-subunit dynamics for substrate capture and positioning. Unlike BSSβ, MASSβ does not interact with the GRD. Instead, MASSδ contacts the GRD, making more extensive contacts than BSSβ (Figure 4C).^77^ Taken together, current structural data suggest that MASSβ gates substrate entry, whereas MASSδ may help control the conformational changes required for glycyl radical installation. However, the specific contributions of the β and δ subunits to radical installation, radical persistence, and catalysis remain to be tested biochemically and clarified by additional structural studies.

The presence of iron cofactors was predicted in BSS quite early on in the characterization of these proteins due to absorbance features between 330 and 450 nm.^71^ Each small subunit of BSS contains conserved cysteine residues which ligate an iron-sulfur cluster. BSSβ, BSSγ, MASSβ, and MASSγ contain [4Fe-4S] clusters coordinated by four cysteine residues (Figure 4F–H). HPADγ contains two [4Fe-4S] clusters, one of which is coordinated by a four-cysteine motif, and the other is coordinated by a three-cysteine one-histidine motif (Figure 4G). MASSδ contains a four-cysteine motif that coordinates a single Fe ion (Figure 4H). The function of the Fe cofactors in GRE small subunits is not well understood. In BSS, the removal of the [4Fe-4S] clusters by a chelating agent caused dissociation of the small subunits from the catalytic subunit, suggesting a role in structural integrity of the small subunits.^53,76^ When the cluster is removed from BSSβ by mutation of one of the coordinating Cys residues, the subunit is extremely prone to proteolysis when expressed in *E. coli*.^76^ HPADγ has been proposed to play a role in glycyl radical reduction as a protective mechanism, although there is no experimental evidence to support this proposal currently.^85^ The [4Fe-4S] clusters found in BSS’s small subunits have similarly been proposed to play a role in electron transfer, again experimental support is currently unavailable.^81^ In short, the role of the iron cofactors in the GRE accessory subunits is enigmatic at present.

### 4.3 GRE Active Site and Substrate Channel

The active sites of GREs are largely comprised of residues located on the strands of the β-barrel in addition to the Gly and Cys loops. GRE active sites are typically specifically tailored to bind and orient the GRE’s native substrate for radical catalysis. As such, GREs typically have a narrow substrate scope and only accept substrates that are close chemical derivatives of their native substrate (Figure 5).

**Figure 5:**
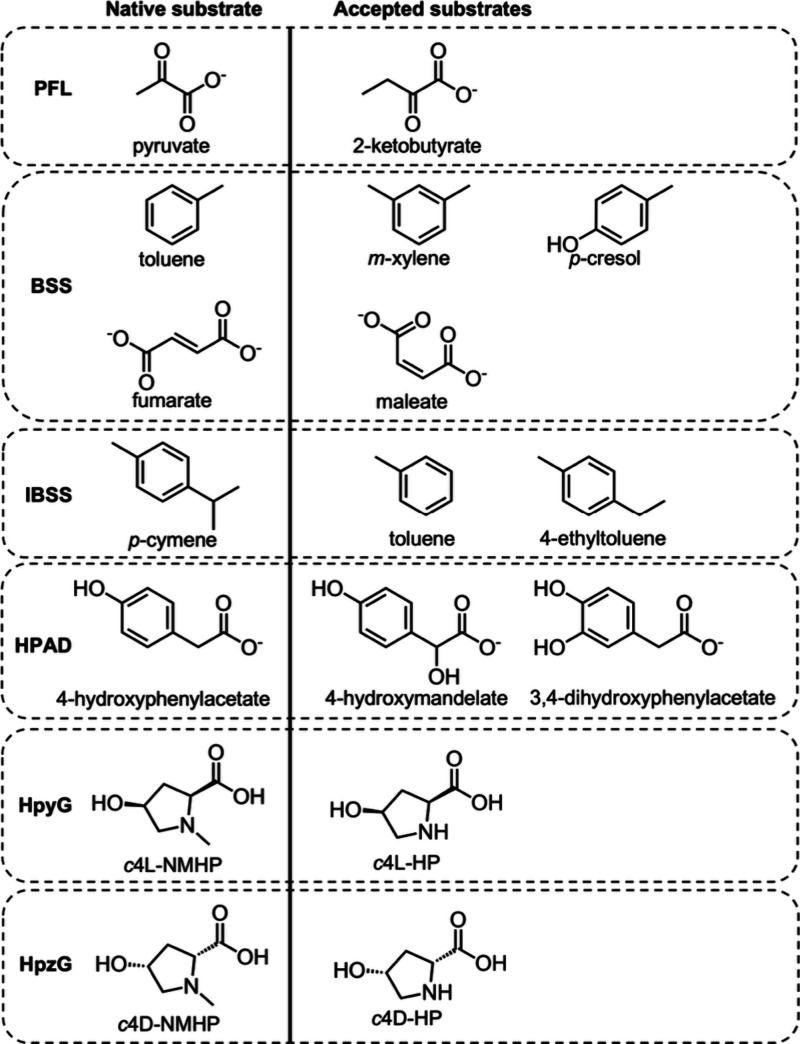
GRE substrate scope. GREs often have limited substrate scope due to their highly tailored active sites. Typically, GREs can only function on their native substrate or close chemical derivatives of their native substrate. The native substrate and other accepted substrates for PFL, BSS, IBSS, HPAD, HpyG, and HpzG are shown.

It is not well understood how GREs regulate entry of substrate to the active site, sequester substrate during the radical-based reaction, and then afford product release. Although all GREs are predicted to have a conserved substrate channel, the substrate channel has been most extensively studied in BSS, which activates the aromatic hydrocarbon toluene through addition to fumarate.^5,84^ In BSS, comparison of αγ and αβγ structures enabled the identification of a substrate channel formed by helices α8′ and α3′ (the cap helix), and a loop following β1 of the barrel (Figure 6A). The cap helix was shown to move inward in BSS upon fumarate binding, and inward even further upon binding of toluene and BSSβ.^84^ In some GREs, residues on the cap helix form hydrogen bonds with the substrate.^37,86,87^

**Figure 6:**
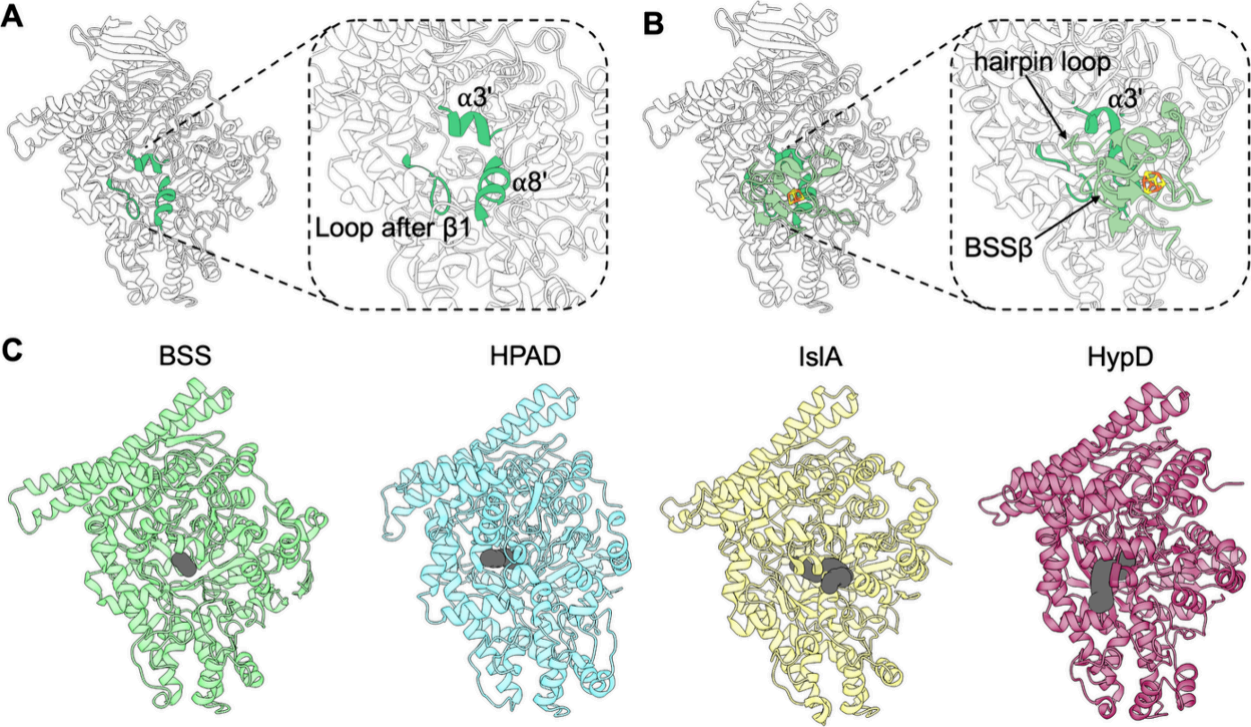
GRE substrate channels. (A) A substrate channel was identified in BSS that is comprised of a loop following β1, α3′, and α8′ (shown in medium sea green) (PDB ID: 5BWE). (B) A hairpin loop of the β subunit (dark sea green) blocks the substrate channel in BSS. (C) The substrate channel in BSS is predicted to be conserved in other GREs (dark gray) and can only be detected by programs such as CAVER in substrate-free enzyme forms: HPAD (PDB ID: 2Y8N), IslA (PDB ID: 3KQ4), and HypD (PDB ID: 6VXC) shown. Predicted channels are shown in gray.

The identified substrate channel in BSS is largely hydrophobic, which is consistent with BSS’s hydrophobic substrate toluene. In the BSSαβγ structure, a hairpin loop of the β subunit is inserted into the active site cavity which leads to this channel, blocking access to it (Figure 6B). As mentioned above, a conserved substrate channel for other GREs has been predicted^84,88^ and is consistent with the channel observed in BSS (Figure 6C). The CAVER-predicted substrate channels could only be detected in GRE structures of HPAD, isethionate sulfite-lyase (IslA), and hydroxyproline dehydratase (HypD) which did not have substrate bound,^88^ suggesting that perhaps the channel does indeed close upon substrate binding in the active site. Sealing the active site would be expected to protect radical intermediate species formed during catalysis. Recently, in the cryo-EM structure of MASS, the predicted substrate channel appears to additionally be blocked by a tryptophan (Trp185 in MASS).^77^ Therefore, glycyl radical enzymes may utilize small subunits, large aromatic residues, substrate binding, or a combination of these factors to regulate substrate access and product release from the active site.

### 4.4 The Conserved Dimerization Interface and Tetrameric Assemblies of GREs

GREs form stable homodimers and have canonically been thought to function as such, but can also form higher order oligomers. By size exclusion chromatography (SEC), GREs can be observed as monomers^87,89^ and dimers^90–92^ and are typically observed as a mix of oligomeric states.^5,77,93^ Certain GREs including HPAD and PFL2, a GRE of unknown function from *Archaeoglobus fulgidus*, form stable tetramers, which can be observed by SEC, small-angle X-ray scattering, and analytical ultracentrifugation.^70,94,95^ Structural studies of GREs have enabled visualization of the dimer and tetramer interfaces of GRE oligomers and demonstrate that the two interfaces are unique from one another.

Of the many experimentally determined GREs structures solved by X-ray crystallography, most show a homodimer of the catalytic subunit within the crystal lattice,^37,86–89,92,94–98^ which has a conserved dimerization interface comprised of α-helices (Figure 7A). The recent structure of MASS solved via cryo-EM also shares this dimerization interface.^77^ Currently, there are three examples of GREs that do not have the conserved dimer within the crystal lattice and instead only show monomers forming the crystal lattice. These structures are glycerol- and 1,6-anhydroglucitol-6-phosphate-bound YbiW and malonate-bound PflD, two GRE sugar isomerases.^39^ No solution state oligomeric information is available for these GREs. For the GRE dimer interface, surface area, solvation free energy upon formation, and complex significance score (CSS) as calculated by PDBePISA are shown in Table 1. The dimer interface surface area ranges between 1000 and 1900 Å^2^ in the GREs for which there are published experimental structures. The CSS scores and energy of solvation vary, suggesting a range of strengths of the conserved dimer interface across GREs.

**Figure 7:**
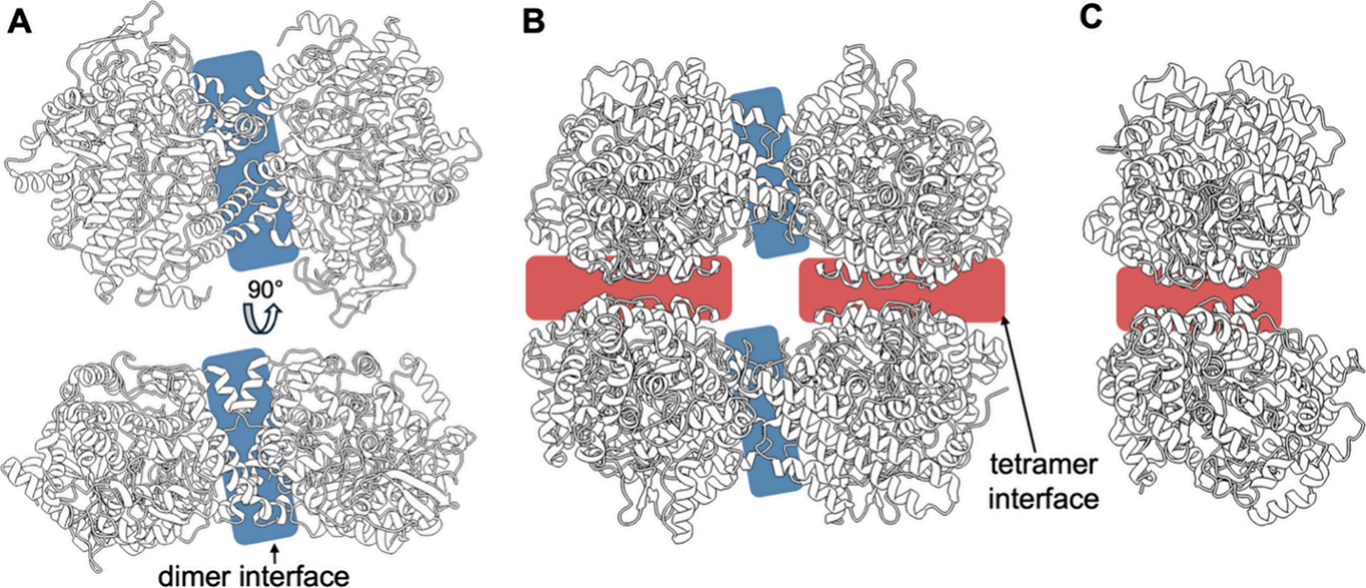
Oligomeric states of GREs. (A) Most GREs solved by X-ray crystallography have a conserved dimer α_2_ interface comprised of α-helices that precede the barrel. PFL is shown here as an example (PDB ID: 1H18). (B) Some GREs, in addition to having the canonical GRE dimer within the crystal lattice, also have a tetrameric assembly of α subunits. HPAD (PDB ID: 2YAJ) is shown here as an example. (C) The GRE isomerase PflD (PDB ID: 8IDO) does not have the canonical α_2_ dimer within its crystal lattice, however, it does have two protomers that align via the tetrameric interface observed in HPAD. Dimer interfaces are indicated by blue box and tetramer interfaces by a red box.

**Table 1:** Area, Solvation Free Energy, and CSS of GRE Homodimer Interfaces

PDB ID	GRE	Area (Å^2^)	solvation free energy upon dimerization (kcal/mol)	CSS (out of 1)
1H18	pyruvate formate lyase (PFL)	1900	–6.0	0.34
5BWE	benzylsuccinate synthase (BSS)	1600	–12.0	1.0
1R9D	glycerol dehydratase (GD)	1200	–8.3	0.66
2YAJ	hydroxyphenylacetate decarboxylase (HPAD)	1800	–15.8	1.0

In addition to the conserved homodimer, some GREs whose structures were solved by X-ray crystallography show a tetrameric assembly within the crystal lattice, despite not forming detectable tetramers in solution. This tetramer is comprised of two GRE homodimers; therefore, any GRE that has the tetramer present also has the canonical GRE dimer present within the crystal lattice. HPAD and PFL2 in addition to being detected as stable α_4_ tetramers in vitro, contain tetramers within their crystal lattices (Figure 7B).^75,95^ The tetramer interface observed in HPAD and PFL2, analyzed with PDBePISA and shown in Table 2, is smaller and weaker than the canonical GRE dimer interface. It is possible the tetrameric GRE assembly may be a more transient oligomeric state in comparison to the GRE dimer. Strangely, the substrate-bound PflD structure does not have the canonical GRE dimer within the crystal lattice, yet does have two monomers aligned by the tetrameric interface observed in HPAD (Figure 7C).^39^ The catalytic relevance of the oligomeric state of GREs has never been directly probed, thus it remains unclear what, if any, correlation exists between structural assemblies of GREs, oligomeric state in vitro, and catalysis.

**Table 2:** Area, Solvation Free Energy, and CSS of GRE Tetramer Interfaces

PDB ID	GRE	area (Å^2^)	solvation free energy upon tetramer formation from two dimers (kcal/mol)	CSS (out of 1)
2YAJ	hydroxyphenylacetate decarboxylase (HPAD)	865	–6.0	0
2F3O	PFL2	1073	+10.2	0
8ID0	PflD^a^	896	–6.3	0

a Note that there is no solution state evidence for the formation of a tetrameric PflD. However, the tetrameric interface seen in other GREs exists within the crystal lattice of PflD and can therefore be analyzed with PDBePISA.

## 5 GRE Classifications and Catalysis

### 5.1 Introduction to GRE Classifications and Catalysis

The GRE superfamily spans two distinct InterPro families, the “anaerobic RNR” (IPR012833) and the “PFL-like” (IPR004184) proteins. All non-RNR GREs, including PFL, fall into the PFL-like division. In other words, being “PFL-like” means that the GRE is not an RNR. It is the “PFL-like” GRE grouping that provides the diversity of reactivity in this superfamily, catalyzing an array of chemical transformations from dehydration to hydrocarbon activation. The enzyme active sites are tailored to each specific chemical transformation by providing residues that stabilize particular conformations of substrate, act as acids or bases, or alter substrate p*K*_a_ and bond strengths. At the time of the last detailed review, the nine types of GREs that were characterized were split into five classes based on function: formate lyases; class III ribonucleotide reductases; 1,2-eliminases; decarboxylases; and X-succinate synthases (Figure 8).^99^ In the subsequent years, many new GRE enzymes have been identified and characterized either structurally, biochemically, or both. The expansion of GRE classes and emerging subclasses has prompted a reevaluation of the categorization of the superfamily. Here, we retain the original five GRE classes and expand the superfamily with two new classes: the GRE isomerases and the aminoacyl radical enzymes (AAREs) (Figure 8). We also further divide the 1,2-eliminases and GRE X-succinate synthases (also known as GRE hydroalkylases) into subclasses based on catalytic function. Finally, we discuss general mechanistic features and active site organization of GREs.

**Figure 8:**
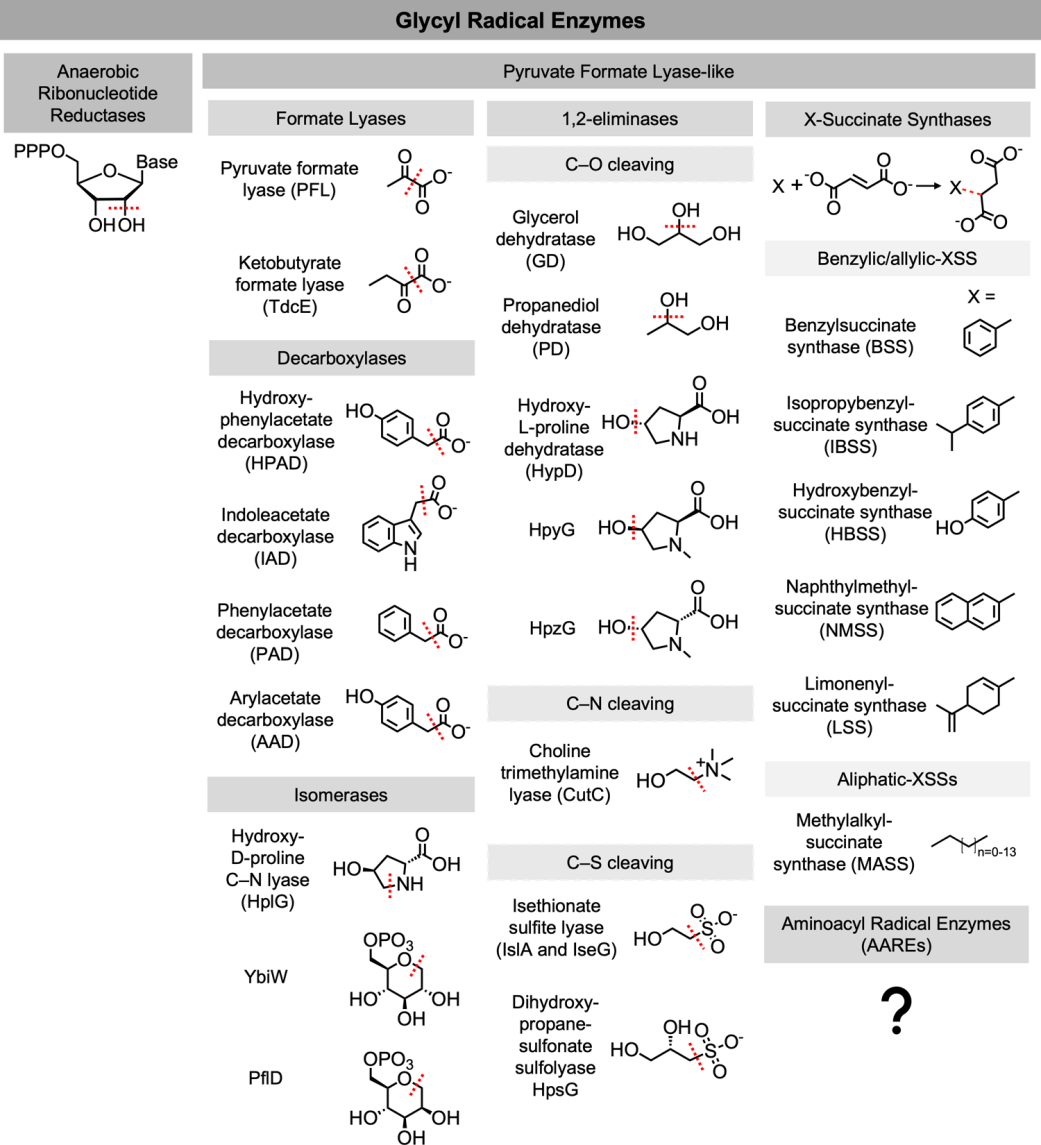
GRE classifications and subclasses and their substrates. Glycyl radical enzymes are divided into seven classes: anaerobic or class III ribonucleotide reductases, formate lyases, decarboxylases, isomerases, 1,2-eliminases, X-succinate synthases, and aminoacyl radical enzymes. 1,2-eliminases and X-succinate synthases are further subclassified. The bond that is cleaved in each substrate or formed in a product is indicated by a dashed red line.

### 5.2 The Anaerobic Class III Ribonucleotide Reductases

The anaerobic class III ribonucleotide reductases, in addition to being a class of glycyl radical enzymes, are a class within the larger ribonucleotide reductase family of enzymes, which contains both aerobic and anaerobic, eukaryotic and prokaryotic enzymes. Within class III RNRs, there are emerging subclassifications. One such classification could be made on the presence or absence of cone domains which play a role in the allosteric activity regulation of the enzyme.^100^ Another could be the mechanism of rereduction of the active site cysteine pair, for example, by formate^101^ or thioredoxin/thioredoxin reductase.^102^ As mentioned above, class III ribonucleotide reductases represent one division of GREs, whereas all other known GREs are referred to as “PFL-like”. As class III RNRs have a plethora of unique structural features, mechanistic steps, and regulatory facets distinct from PFL-like GREs, we will not be focusing on the anaerobic RNRs here. They have been reviewed extensively elsewhere.^100,103,104^

### 5.3 Formate Lyases

Currently, there are only two known types of formate lyases, pyruvate formate lyase (PFL) and 2-ketobutyrate formate lyase (TdcE). PFL and TdcE catalyze the C–C bond cleavage of pyruvate and 2-ketobutyrate, respectively, followed by the thioester bond formation to produce acetyl-CoA or propionyl-CoA, with formate generated as a side product. PFL was the first identified GRE,^2^ and based on current sequencing and polypeptide annotations, is the most widespread GRE in microorganisms, likely due to its role in anaerobic primary metabolism.^105–107^ PFL has consequently been particularly well studied and has become the namesake for the non-RNR glycyl radical enzymes: the PFL-like GREs. Uniquely, PFL’s mechanism is split into two distinct half reactions where a stable acyl-enzyme intermediate is generated following the first half reaction.^1^ The second half reaction forms acetyl-CoA, which can be metabolized to acetate coupled to the generation of ATP.^108^

Oxamate-, pyruvate-, and CoA-bound X-ray structures of PFL combined with biochemical work have illuminated aspects of the catalytic mechanism of PFL.^1,7,18,49^ PFL and TdcE are the only non-RNR GREs that encode two cysteines on the Cys loop (C418 and C419 in *Ec*PFL), both of which are catalytically essential.^42,109,110^ A thiyl radical is first generated at Cys419 and then transferred to Cys418 (Figure 9A). Unlike most other GRE mechanisms, the Cys418 thiyl radical adds to the carbonyl of pyruvate, generating a tetrahedral intermediate that can rapidly eliminate a formyl radical and acetylated Cys418. It is proposed that this formyl radical abstracts an H-atom from Cys419 (Figure 9A). Formate can leave the active site as CoA enters, though there is currently no structure available of CoA bound within the PFL active site. The Cys419 thiyl radical abstracts the thiol hydrogen of CoA to form a CoA thiyl radical, which can add to the carbonyl carbon of the acetyl group on Cys418 (Figure 9A). The tetrahedral intermediate collapses to release acetyl-CoA and the thiyl radical at Cys418, where it can move back to Gly734 for storage (Figure 9A). In the pyruvate-bound structure of PFL, Arg435 and Arg176 form hydrogen bonds with pyruvate and likely play a key role in stabilization of its conformation in the active site (Figure 9B). In the pyruvate and CoA-bound crystal structure, CoA is not bound in a catalytically relevant fashion but rather sits outside the predicted substrate channel in PFL (Figure 9C). Interestingly, the adenine moiety of CoA is stabilized by Asn145 and Gln146 in a small binding pocket outside the predicted substrate channel, and the authors note that rotation of the ribose-pantetheine moiety around the *N*-glycosidic bond could provide a model of CoA bound in a catalytically relevant fashion where the cysteamine tail of CoA presumably extends into the substrate channel of PFL.^1^ Another open question is how the cell protects itself from the formate produced by the PFL reaction. One possibility is that formate build-up within the cell is prevented by an association of PFL with the formate translocation channel FocA.^111^ Another possibility is that formate is consumed by reaction of other enzymes such as a formate-dependent class III RNR.^102^

**Figure 9:**
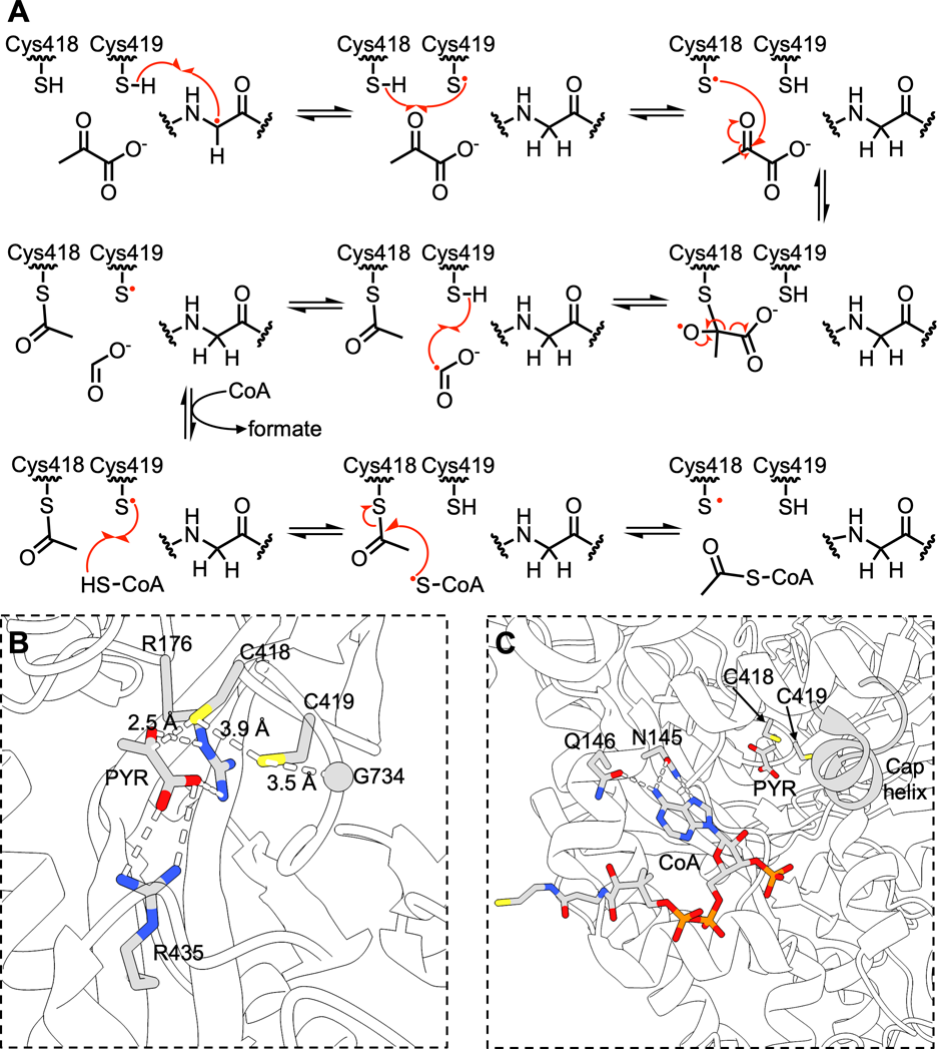
GRE formate lyase structure and mechanism. (A) Summary of the proposed PFL mechanism. (B) Pyruvate formate lyase structure (PDB ID: 1H16) showing pyruvate (PYR) binding site. Distances from Gly734 to Cys419 to Cys418 to PYR–C2 are shown. (C) Pyruvate formate lyase structure showing CoA binding site near predicted substrate channel.

The formate lyases are the only class of GREs for which small “spare-part” proteins have been identified. In *E. coli*, this protein is termed YfiD, also referred to as autonomous glycyl radical cofactor, GrcA.^112^ YfiD contains a disordered N-terminus and a C-terminus that structurally resembles the C-terminal GRD of PFL that houses the glycyl radical cofactor.^113,114^ The glycyl radical, as discussed above, is extremely oxygen sensitive.^11^ Exposure to oxygen results in cleavage of the polypeptide backbone at the glycyl radical site, rendering the enzyme inactive.^11,49^ In what likely evolved as a protective mechanism, YfiD can restore function to PFL following oxygen-induced cleavage of the C-terminal GRD.^50,112^ PFL-AE can install a glycyl radical on the C-terminal GRD of YfiD and then the YfiD can take the place of the damaged part of PFL. Interestingly, the C-terminus of YfiD’s GRD includes a β-strand that overlaps with a C-terminal β-strand retained in oxygen-cleaved PFL. Although this overlapping strand was originally proposed to be removed before YfiD binding,^113^ biochemical studies showed that YfiD binds oxygen-cleaved PFL without further truncation. Instead, removing this region from PFL prevents efficient YfiD-mediated rescue. Whereas the function of the C-terminal region of YfiD is clear; its GRD replaces the cleaved PFL GRD, the function of the disordered N-terminal region of YfiD has been enigmatic. We know that removal of the disordered N-terminal region of YfiD leads to decreased glycyl radical installation by PFL-AE, but that the N-terminus is not required for catalysis once the radical has been installed.^115^ It is yet to be determined if spare-part proteins exist for GREs aside from PFL.

### 5.4 GRE Decarboxylases

GRE decarboxylases catalyze the cleavage of a C–C bond between the carboxylate moiety and the aryl group of the substrates 4-hydroxyphenylacetate (4HP), indole-3-acetate (I3A), and phenylacetate (PA) (Figure 8). There are four currently identified GRE decarboxylases: hydroxyphenylacetate decarboxylase (HPAD),^83^ phenylacetate decarboxylase (PAD),^116^ indoleacetate decarboxylase (IAD),^33^ and arylacetate decarboxylase (AAD)^89^ (Figure 10A). HPAD was the first GRE decarboxylase to be identified and catalyzes the conversion of 4HP to form *p*-cresol. HPAD is proposed to use a Kolbe-type decarboxylation mechanism (Figure 10B) instead of the hydrogen atom transfer (HAT) mechanism canonically employed by GREs.^75,99^ The Kolbe-type mechanism for HPAD was proposed based on the X-ray crystal structure, which showed an unexpected substrate binding mode where the carboxylate moiety of 4HP was oriented near the catalytic Cys503 (Figure 10C).^75^ In the HPAD active site, Glu505 and Glu637 hydrogen bond with 4HP and are proposed to play catalytically essential roles (Figure 10D).^75^ The proposed Kolbe-type mechanism begins with a one-electron oxidation of the substrate carboxylate by the thiyl radical, resulting in a cysteine thiolate and a substrate radical (Figure 10B). Concerted deprotonation of the substrate phenol by Glu637 is thought to stabilize the corresponding radical that can then undergo spontaneous decarboxylation to form a benzylic radical. Finally, protonation of Cys503 by Glu505 reforms the thiol of Cys, and reabstraction of this H atom by the product radical yields *p*-cresol and regenerates the thiyl radical (Figure 10B). Mutagenesis has not been performed to confirm the necessity of these residues for catalysis, though the mechanism has been supported by extensive computational work.^117^

**Figure 10:**
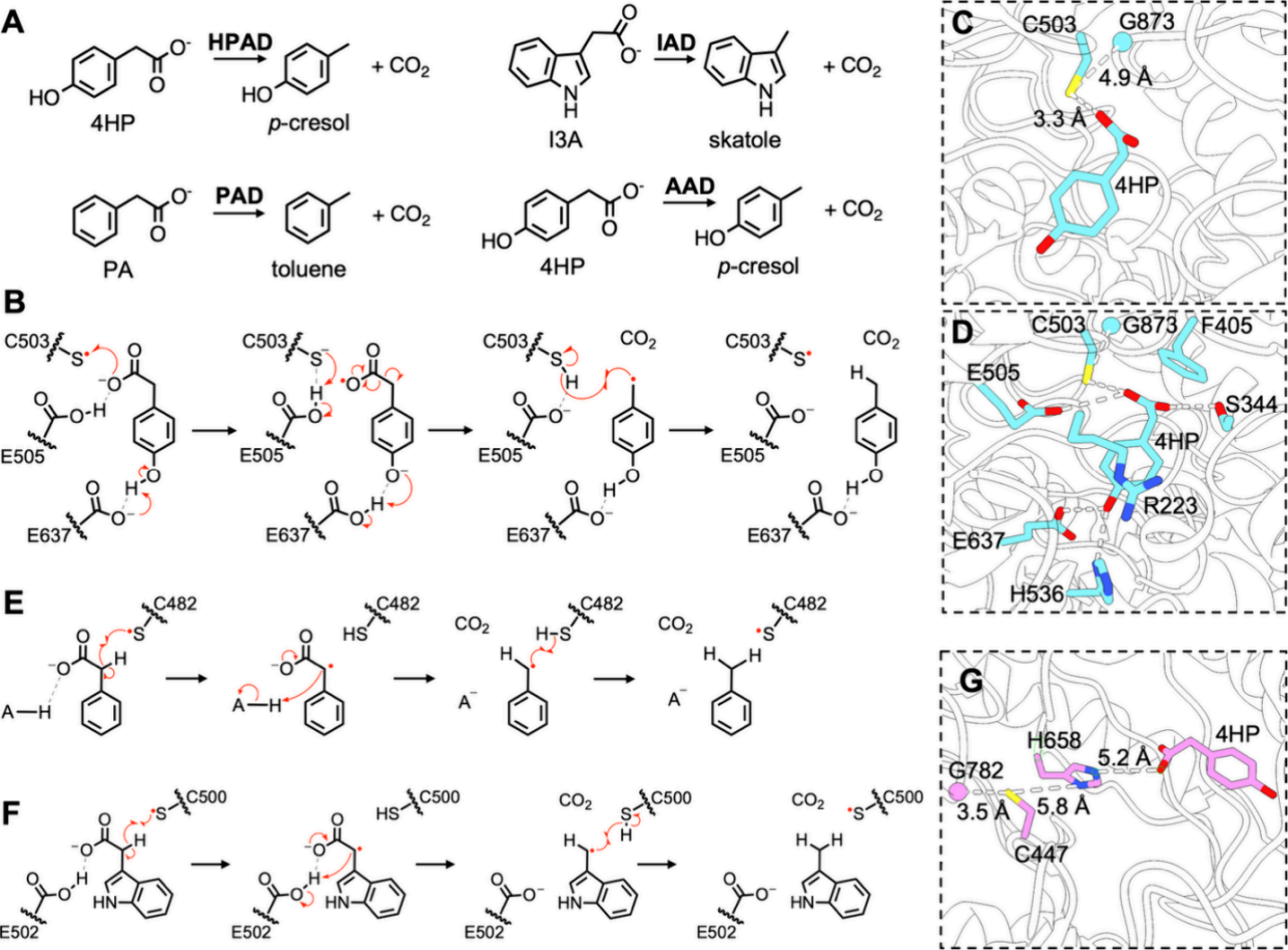
GRE decarboxylase structure and mechanism. (A) Schemes showing reactions of the GRE decarboxylases. (B) Proposed Kolbe-type mechanism for HPAD. (C) HPAD structure (PDB ID: 2YAJ) showing 4HP binding mode where the carboxylate of 4HP is oriented near the catalytic Cys503. (D) HPAD structure (PDB ID: 2YAJ) showing overall active site architecture of HPAD. (E) Proposed HAT mechanism for PAD. (F) Proposed HAT mechanism for IAD. (G) AAD structure shows substrate 4HP far away from the active site (11 Å distance between 4HP and the catalytic Cys) in what may be a substrate channel (PDB ID: 7E7L). No structure with the substrate positioned for catalysis is available.

PAD and IAD were the next additions to the GRE decarboxylase class and catalyze the conversion of phenylacetate (PA) to form toluene, and indole-3-acetate (I3A) to form skatole, respectively (Figure 10A).^33,116^ PAD and IAD have been proposed to act via the canonical HAT mechanism used by GREs.^91,93^ By this mechanism, the thiyl radical abstracts a hydrogen atom from the carbon alpha to the carboxylate of each substrate as the first step in catalysis (Figure 10E,F). This mechanism would require a proton donor to protonate the α-carbon to facilitate decarboxylation, thus generating a benzylic radical. This radical reabstracts an H atom from the catalytic Cys residue to form the thiyl radical. In PAD, the required acidic residue is unknown. In IAD, Glu502 has been proposed to act as the proton donor (Figure 10E,F).^93^ Mechanistic proposals for PAD and IAD were based on biochemical studies in the absence of experimentally solved structures. In PAD, an HAT mechanism was proposed because fluorination of the α-carbon of PA inhibited enzyme activity.^91^ Mutagenesis studies showed that Tyr691, Gln484, and Trp495 in PAD are catalytically essential.^91^ In IAD, isotopic labeling studies were leveraged to probe IAD’s mechanism of catalysis. Reactions of activated IAD with its substrate I3A conducted in D_2_O showed a deuterium incorporation pattern in skatole that seemed more consistent with a HAT mechanism over a Kolbe-type mechanism.^93^ Further, mutagenesis demonstrated that Glu502, Arg226, Leu616, Phe401, and Trp392 are essential for catalysis.^93^ However, without experimental structures of PAD and IAD, it is challenging to propose molecular mechanistic explanations for the necessity of each residue identified as important via mutagenesis studies.

The most recent GRE decarboxylase to be identified is a unique 4-hydroxyphenylacetate decarboxylase that is termed arylacetate decarboxylase (AAD).^89^ AAD, like HPAD, converts 4HP to *p-*cresol. Interestingly, 4-aminophenylacetate is also tolerated as a substrate. Beyond different substrate promiscuities, two additional distinctions between HPAD and AAD are observed: active site residues are not conserved between the two enzymes and AAD does not use an accessory subunit like HPAD.^75,89^ An X-ray crystal structure of AAD has been solved and shows the substrate 4HP bound in a noncatalytically relevant position over 11 Å away from the catalytic Cys447 (Figure 10G).^89^ Surprisingly, despite both enzymes catalyzing decarboxylation of 4HP, there are few similarities in the active site architecture of HPAD and AAD. It should be noted, however, that the resolutions of these structures are quite different. 4HP-bound HPAD is at 1.8 Å resolution, whereas 4HP-bound AAD is at 3.53 Å resolution. Moreover, the model refinement *R*free factor for this AAD structure, which is a metric of model-to-data agreement, is very high (0.358; PDB ID = 7E7L), even compared to structures of the same moderately low resolution. This high *R*free factor suggests poor agreement between the model and the experimental data. Unfortunately, the poor model quality statistics cast doubt on the structure’s validity and limit the mechanistic information obtainable from the presented model. Overall, it is unclear why four enzymes that perform similar chemical reactions would use distinct mechanisms of catalysis. More work will surely be necessary to fully understand this discrepancy.

### 5.5 GRE X-Succinate Synthases/GRE Hydroalkylases

The activation of hydrocarbons via addition to fumarate is catalyzed by GRE X-succinate synthases (XSSs).^4,65,72,74,118–124^ These enzymes form C­(sp^3^)–C­(sp^3^) bonds between their hydrocarbon substrates and fumarate through hydroalkylation (Figure 8) and are thus also known as GRE hydroalkylases. The best-characterized XSS is BSS, which stereoselectively forms (*R*)-benzylsuccinate through hydroalkylation of fumarate with toluene.^71,125–129^ Biochemical work, computation, and structural data are all consistent with the activation of toluene occurring at the methyl carbon (Figure 11B).^84,126,128–133^ After BSS performs H-atom abstraction from a C–H bond of the methyl group of toluene, the resulting benzylic radical adds to fumarate to form a benzylsuccinyl radical. The benzylsuccinyl radical reabstracts H atom from the catalytic Cys to form product and regenerate thiyl radical (Figure 11A).^126,129–132,134^ (*R*)-benzylsuccinate can be subsequently oxidized to CO_2_, enabling bacterial growth on toluene as a sole carbon source.^135–139^

**Figure 11:**
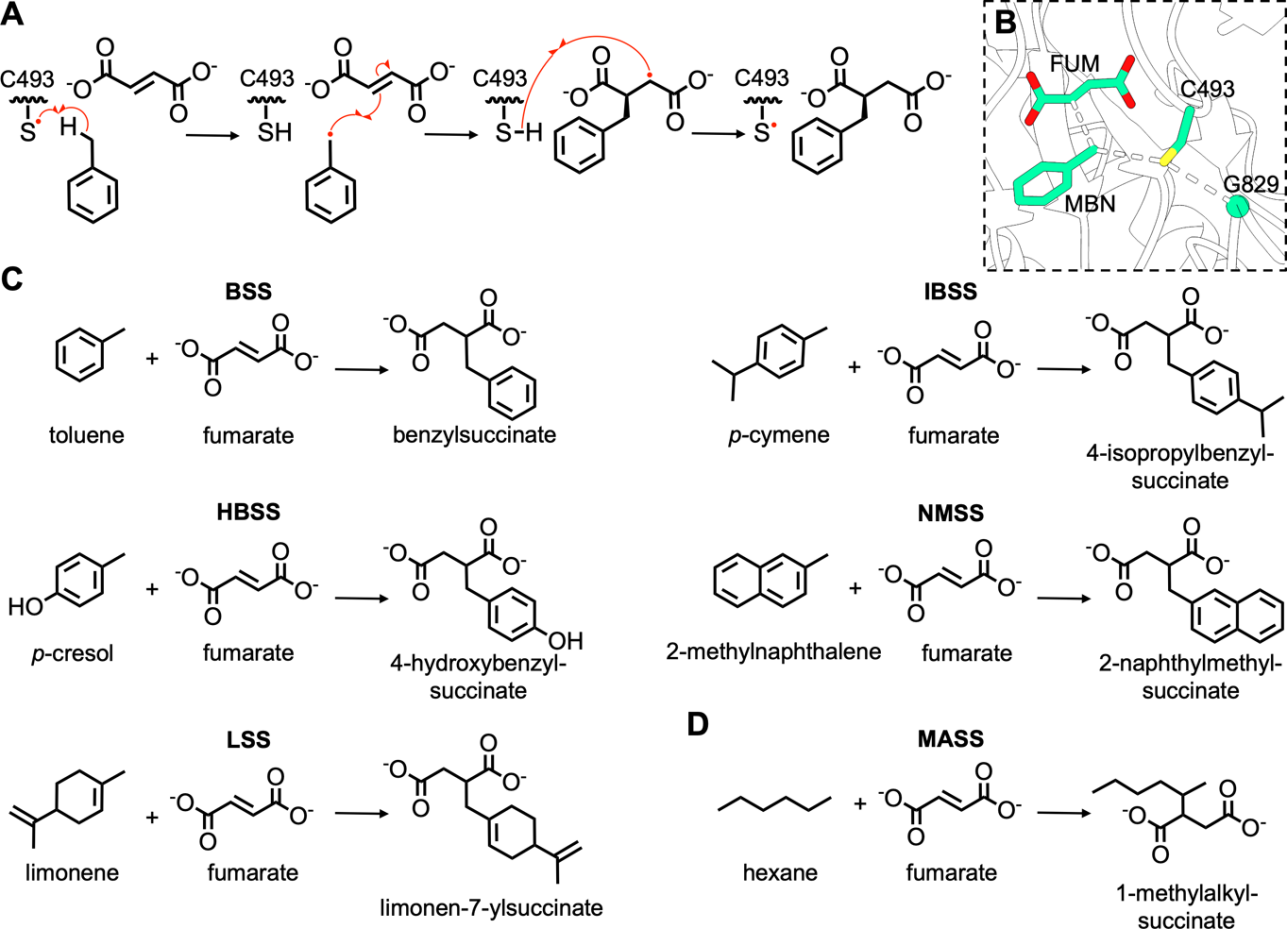
GRE X-succinate synthase structure, mechanism, and reaction schemes. (A) Reaction scheme for BSS. Only the hydrogen that is transferred is shown. (B) BSS structure (PDB ID: 5BWE) showing active site where fumarate (FUM) and toluene (MBN) bind near Cys493. (C) Benzylic/allylic-XSS reaction schemes. (D) Aliphatic-XSS reaction schemes.

#### 5.5.1 Benzylic/Allylic Succinate Synthases

Since the discovery of BSS, numerous additional unique XSS enzymes have been identified that function on a range of aromatic, allylic, and aliphatic hydrocarbon substrates. The expansion of XSSs has motivated our proposal of splitting the class into two subclasses: arylalkyl-XSSs (benzylic/allylic-SSs) and alkyl-XSSs (aliphatic-SSs) (Figure 8). The arylalkyl-XSSs include BSS; isopropylbenzylsuccinate synthase (IBSS), which adds *p*-cymene to fumarate to form 4-isopropylbenzylsuccinate;^140^ hydroxybenzylsuccinate synthase (HBSS) which adds *p*-cresol to fumarate to form 4-hydroxybenzylsuccinate;^141^ naphthyl-2-methylsuccinate synthase (NMSS) which adds 2-methylnaphthalene to fumarate to form 2-methylnaphthylsuccinate;^142,143^ and limonenylsuccinate synthase (LSS) which adds limonene to fumarate to form limonen-7-ylsuccinate (Figure 11C).^144^ Notably, LSS acts on an allylic substrate rather than a benzylic one. However, because its sequence homology, subunit architecture, and the C–H bond dissociation energy at the abstracted position are all more similar to BSS than to aliphatic-SSs, we classify LSS within the benzylic/allylic subclass. Within this subclass, so far, there is conservation of the αβγ architecture observed in BSS, and it is yet to be determined if benzylic/allylic-SSs with varying numbers of subunits exist.

#### 5.5.2 Aliphatic Succinate Synthases

The alkyl-succinate synthases function on a range of *n-*alkanes (C_3_ to C_16_).^72,74,145–148^ 1-methylalkyl-succinate synthase or MASS activates *n*-hexane by adding it to fumarate to form (1-methylpentyl)­succinate (Figure 11D).^147^ The MASS from Azoarcus sp. HxN1 has recently been structurally characterized.^77^ Investigations into the stereochemistry of the product by MASS showed a mixture of diastereomers formed; however, these assays were conducted within the native organism.^149^ As such, the authors propose that the lack of stereoselectivity at one stereocenter arises from a downstream epimerase. Further studies will be necessary to confirm the stereochemistry of the product. As anaerobes are known to grow on a variety of hydrocarbon substrates,^150^ it is not difficult to imagine that additional GREs are yet to be discovered, which will expand each subclass of XSSs.

### 5.6 GRE 1,2-Eliminases

The GRE 1,2-eliminases are the most rapidly expanding class of GREs. Since the last comprehensive review in 2017,^99^ six new GRE eliminases have been identified and characterized in depth. In addition to new C–O bond cleaving eliminases, C–S bond cleaving eliminases have also been discovered in the past decade. Thus, we subclassify GRE 1,2-eliminases based on the type of bond cleavage they perform: C–O, C–N, or C–S (Figure 8).

#### 5.6.1 C–O Cleaving Eliminases

First, we begin with a summary of the C–O bond-cleaving GRE eliminases. There are now six total C–O bond cleaving eliminases: glycerol dehydratase (GD) catalyzes the dehydration of glycerol (GOL) to form 3-hydroxypropionaldehyde (Figure 12A);^151^ propanediol dehydratase (PD) catalyzes the dehydration of propane-1,2-diol (PDO) to propionaldehyde (Figure 12B);^152^ dihydroxypropanesulfonate dehydratase (HpfG) catalyzes the dehydration of 2­(*S*)-dihydroxypropanesulfonate (DHPS) to form 3-sulfopropionaldehyde;^86^ hydroxyproline dehydratase (HypD) acts on *trans*-4-hydroxy-L-proline (t4L-HP), dehydrating it to form pyrroline-5-carboxylate (Figure 12C);^105^ HypG catalyzes the dehydration of N-methyl-*cis*-4-hydroxy-L-proline (*c*4L-NMHP) to form N-methyl-pyrroline-5-carboxylate (Figure 12D);^92^ HpzG catalyzes the dehydration of N-methyl-*cis*-4-hydroxy-D-proline (*c*4D-NMHP) to form N-methyl-pyrroline-5-carboxylate (Figure 12E).^92^

**Figure 12:**
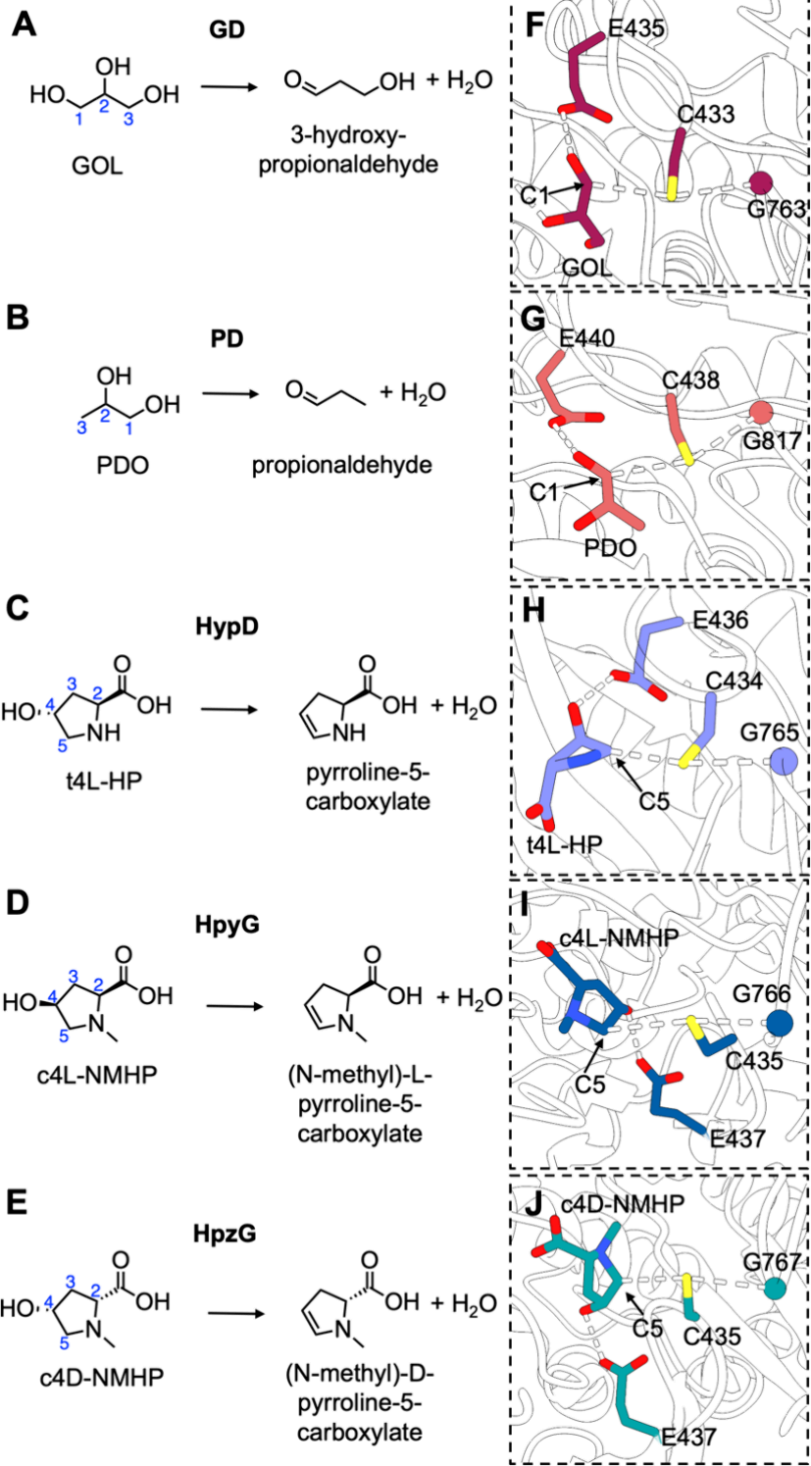
GRE 1,2-eliminase structure and schemes. (A) Reaction scheme for GD. (B) Reaction scheme for PD. (C) Reaction scheme for HypD. (D) Reaction scheme for HpyG. (E) Reaction scheme for HpzG. (F) Structure of GD (PDB ID: 1R9D) showing GOL (glycerol) bound. (G) Structure of PD (PDB ID: 5I2G) showing PDO (1,2-propanediol) bound. (H) Structure of HypD (PDB ID: 6VXE) showing t4L-HP (*trans*-4-hydroxy-L-proline) bound. (I) Structure of HpyG (PDB ID: 8ID1) showing c4L-NMHP (N-methyl *cis*-4-hydroxy-L-proline) bound. (J) Structure of HpzG (PDB ID: 8IOF) showing c4D-NMHP (N-methyl *cis*-4-hydroxy-D-proline) bound.

All six of these enzymes retain a CXE motif on the loop containing the catalytic cysteine. In all cases where experimental structures are available, the catalytic cysteine is positioned near the site of proposed HAT, and the glutamate of the CXE motif is hydrogen bonded to substrate. In GD, Cys433 is positioned near C1 of GOL and Glu435 hydrogen bonds to GOL (Figure 12F);^97^ in PD, Cys438 is positioned near C1 of PDO, and Glu440 is hydrogen bonded to PDO (Figure 12G);^94^ in HypD, Cys434 is positioned near C5 of *t*4L-HP and Glu436 is hydrogen bonded to *t*4L-HP (Figure 12H);^37^ and in both HpyG and HpzG, Cys435 of each enzyme is oriented near C5 of the respective substrates, *c*4L-NMHP or *c*4D-NMHP, and Glu437 is hydrogen bonded to the substrates (Figure 12I,J).^92^ In HypD, HpyG, and HpzG, the hydroxyl group that is hydrogen bonded to the glutamate residue leaves as water following the initial HAT from the appropriate C atom.^37,92^ In HypD, HpyG, and HpzG, the hydrogen atom initially abstracted from C5 is proposed to be returned to C4, which has been confirmed by isotopic labeling studies in HypD.^37^ Originally, GD was proposed to use a migration mechanism that was later shown to be unlikely, as a direct elimination is quite feasible.^97,153,154^ An updated mechanism has been proposed, whereby the C1–OH of glycerol is deprotonated by Glu435, while His164 protonates the C2 hydroxyl to promote loss of water.^153^ The resulting product radical reabstracts the cysteine thiol hydrogen atom, forming 3-hydroxylpropionaldehyde. For PD, the structure alone seems to allow for either a 1,2-migration or a direct elimination,^94^ and more work will be necessary to clarify the mechanism. There is no experimental structure available for HpfG, however, homology modeling shows a predicted substrate binding mode similar to that of GOL in GD.^86^

#### 5.6.2 C–N Cleaving Eliminases

Choline trimethylamine lyase (CutC), which cleaves choline (CHT) to form trimethylamine (TMA) and acetaldehyde (Figure 13A), remains the only known C–N bond cleaving 1,2-eliminase.^90^ Trimethylamine produced by CutC in gut microbes can be further oxidized by flavin-containing monooxygenase (FMO) to trimethylamine oxide (TMAO),^155^ which has been implicated in cardiovascular disease.^156^ In the crystal structure of CutC, the catalytic Cys489 is oriented toward C1 of choline, the site of HAT (Figure 13B).^96^

**Figure 13:**
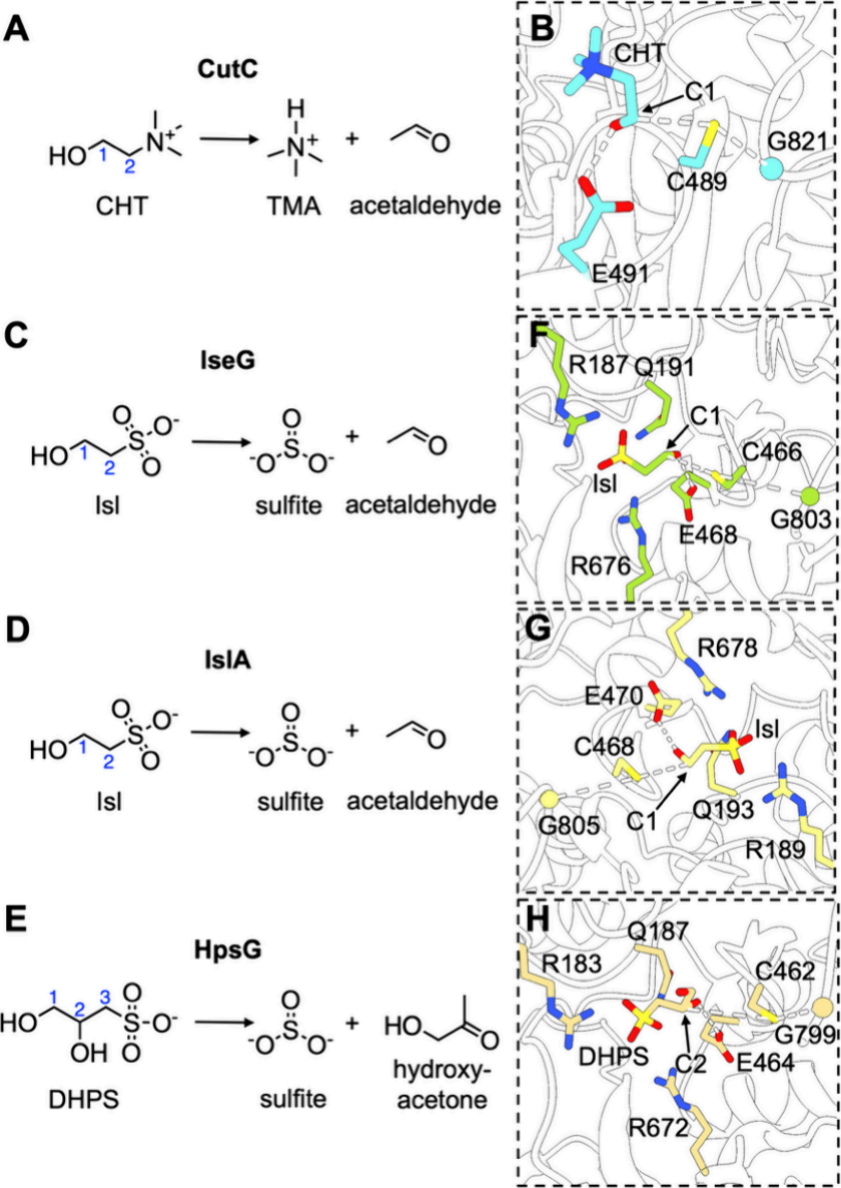
GRE 1,2-eliminase structure and schemes. (A) Reaction scheme for CutC. (B) Structure of CutC (PDB ID: 5FAU) showing choline (CHT) bound. (C) Reaction scheme for IseG. (D) Reaction scheme for IslA. (E) Reaction scheme for HpsG. (F) Structure of IseG (PDB ID: 5YMR) showing Isl (isethionate) bound. (G) Structure of IslA (PDB ID: 7KQ3) showing Isl bound. (H) Structure of HpsG (PDB ID: 6LON) showing DHPS (2-dihydroxypropanesulfonate) bound.

Similar to the C–O bond cleaving GREs, the glutamate of the CXE motif (Glu491 in CutC) hydrogen bonds to the hydroxyl group of choline. Initial hydrogen atom abstraction from C1 is followed by deprotonation of the substrate hydroxyl by the Glu491, facilitating heterolytic bond cleavage of the nitrogen-based leaving group. The resulting acetaldehyde-centered radical abstracts an H atom from Cys489 to form acetaldehyde and generate the thiyl radical. As expected, Glu491 was found via mutagenesis studies to be catalytically essential.^96^

#### 5.6.3 C–S Cleaving Eliminases

The third subclass of 1,2-eliminases are the C–S bond cleaving GREs: isethionate sulfite lyases (IslA and IseG) and 2-hydroxypropanesulfonate sulfolyase (HpsG). These enzymes act on isethionate (Isl)^98,157^ or 2-dihydroxypropanesulfonate (DHPS),^158^ respectively, to form sulfite and acetaldehyde or hydroxyacetone (Figure 13C–E).^159^ The sulfite produced in these reactions can be further reduced to H_2_S, a harmful metabolite implicated in gut disease.^159^ Each C–S bond cleaving GRE also has a published experimental structure,^88,98,158^ allowing for a comparison of three highly similar enzymes. In the active site of IseG (Figure 13F) and IslA (Figure 13G) the substrates are each bound with C1 oriented near the catalytic cysteine (Cys466 in IseG and Cys468 in IslA) and the substrate hydroxyl hydrogen bonding to the glutamate of the CXE motif (Glu468 in IseG and Glu470 in IslA).^88,98^ In HpsG, the C2 of DHPS is near Cys462, and a substrate hydroxyl is hydrogen bonding to Glu464 (Figure 13H).^158^ Interestingly, each C–S lyase uses two arginines and a glutamine residue to anchor the sulfite moiety in the active site. The corresponding residues are Arg183, Gln187, and Arg672 in HpsG; Arg187, Gln191, and Arg676 in IseG; and Arg189, Gln193, and Arg678 in IslA.

The mechanism for C–S bond cleavage is similar to that of CutC. The first step is thought to be the canonical HAT utilized by GREs. IslA is proposed to act via a direct elimination, in which deprotonation of the substrate hydroxyl by Glu470 results in a ketyl radical species that readily decomposes, resulting in heterolytic C–S bond cleavage.^88^ Reabstraction of the hydrogen from the catalytic cysteine is a common step in all reactions. In IslA, Glu470, Gln193, Arg189, Arg678, and V680 are found to be catalytically essential in mutagenesis studies.^88^ A mechanism for IseG is not proposed; however, due to its high similarity to IslA, it is reasonable to hypothesize the mechanisms would be quite similar. In HpsG, the deprotonation of the C2-OH is proposed to occur after dissimilation of substrate to release sulfite, forming an α-keto radical that is then quenched by Cys462.^158^

### 5.7 GRE Isomerases

Three enzymes that establish a new isomerase class of GREs have recently been biochemically and structurally characterized.^39,87^ HplG catalyzes the C–N bond cleavage of *trans*-4-hydroxy-D-proline (*t*4D-HP) to form 2-amino-4-ketopentanoate (Figure 14A).^87^ YbiW and PflD catalyze the C–O bond cleavage of two sugars, 1,5-anhydroglucitol-6-phosphate (AG6P) and 1,5-anyhydromannitol-6-phosphate (AM6P), respectively, to form 1-deoxyfructose-6-phosphate (DF6P)^39^ (Figure 14B,C). DF6P can then be cleaved by downstream aldolases to form glyceraldehyde-3-phosphate and hydroxyacetone, which can be fed into central metabolism.^39,160^

**Figure 14:**
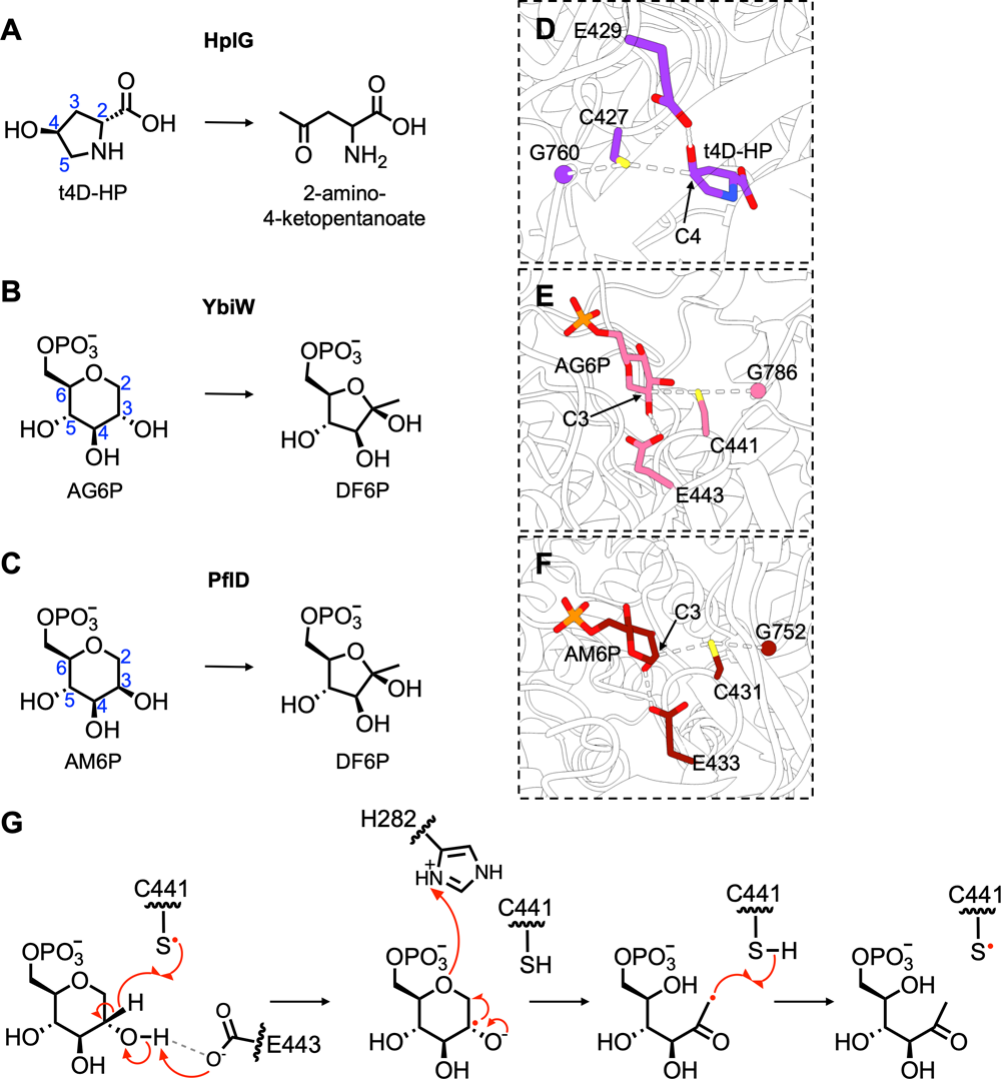
GRE isomerase structure and mechanism. (A) HplG reaction scheme. (B) YbiW reaction scheme. (C) PflD reaction scheme. (D) HplG (PDB ID: 7VUA) showing t4D-HP binding mode where C4 of t4D-HP is near Cys427 and Glu429 is hydrogen bonding to C4-OH. (E) YbiW (PDB ID: 8ID7) showing AG6P binding mode where C2 of AG6P is near Cys441 and Glu443 is hydrogen bonding to C2-OH. (F) PflD (PDB ID: 8ID0) showing AM6P binding mode where C2 of AM6P is near Cys431 and Glu433 is hydrogen bonding to C2-OH. (G) Reaction mechanism for YbiW.

Crystal structures of all three enzymes have been solved,^39,87^ showing active site architecture and lending insight into the enzyme’s mechanisms of catalysis. All proposed mechanisms begin with HAT from substrate by the thiyl radical. Cys427 in HplG is near C4 of *t*4D-HP (Figure 14D); Cys441 in YbiW is near C2 of AG6P (Figure 14E); and Cys431 in PflD is near C2 of AM6P (Figure 14F).^39,87^ Similarly to the GRE 1,2-eliminases, the GRE isomerases retain the CXE motif on the Cys loop, where the glutamate hydrogen bonds with substrate (Glu429 in HplG; Glu443 in YbiW; and Glu433 in PflD) (Figure 14D–F).^39,87^ In YbiW and PflD, initial HAT is followed by deprotonation of the C2–OH by the glutamate residues. Deprotonation likely facilitates heterolytic C–O bond cleavage within the ring and the formation of an α-keto radical, which can then be converted to product by HAT from the respective cysteine thiols.^39^ An example of this mechanism for YbiW is given in (Figure 14G). The GRE isomerases share a similarity with other GREs in that they catalyze a C–O or C–N bond cleavage reaction; however, they are distinct in that their products are simply isomers of the substrate, whereas GRE 1,2-eliminases perform net elimination to form an unsaturated product.

### 5.8 Aminoacyl Radical Enzymes (AAREs)

An unprecedented expansion of the GRE superfamily was presented in 2024 with the discovery of glycyl radical-like polypeptide sequences in metagenomic data that do not contain the conserved glycine seen in GREs but rather a threonine, serine, or alanine residue.^47^ These threonyl-, seranyl-, and alanyl-radical enzymes, or aminoacyl radical enzymes (AAREs), have been confirmed experimentally and over 100 homologues have been identified bioinformatically.^47^ The AAREs have high sequence similarity to known GREs, cluster with GREs phylogenetically, and have predicted folds highly similar to GREs.^47^ Thus, AAREs seem to fall under the PFL-like division of GREs. The existence of highly similar yet nonglycyl radical enzymes challenges many long-held beliefs in the field of protein-based radical enzymes. No functions have been elucidated for AAREs to date, and there is much to be discovered about this interesting new branch of the GRE superfamily.

## 6 GRE Cellular Compartments

In the past decade, bioinformatic studies have unveiled multiple examples of GREs that are associated with cellular compartmentalization in bacteria. Bacterial microcompartments (BMCs) encapsulate key metabolic pathways within protein-based, icosahedral shells (typically ranging from 100 to 400 nm),^161^ selectively allowing the entrance and exit of substrates and products to generate ATP. Because BMC shell proteins and associated enzymes are typically encoded together within the same genetic locus, BMC loci are readily identified and their functions predicted based on characterized associated enzymes, although many remain of unknown function. With the rise of microbiome sequencing and genome-resolved metagenomic advances, the number of identified BMCs has rapidly increased; BMCs have been identified across 45 bacterial phyla with 68 distinct types, and more are still being discovered, thus expanding their functional range.^162^

These bioinformatic advances revealed that the most abundant class of metabolic BMCs are glycyl radical enzyme microcompartments (GRMs), which encapsulate the signature GRE and downstream metabolic enzymes.^163^ Thus far, eleven distinct GRM types and subtypes have been annotated, depending on their protein shell composition and the enzymes encapsulated in them.^162^ These GRMs can be categorized within the following classes proposed by Zarzycki et al. in 2015:^163^ GRM1 and GRM2 are associated with CutC and choline metabolism; GRM3, GRM4, and GRM6 are associated with PD and 1,2-propanediol metabolism; GRM5 is associated with rhamnose and fucose metabolism; and GRMguf is associated with IslA and isethionate metabolism. The latter is referred to as “GREguf” because it was initially a distinct GRM class associated with a GRE of unknown function. In 2021, its association with the GRE IslA was determined by Burrichter et al.^164^ Interestingly, the downstream product of rhamnose and fucose metabolism associated with GRM5 is 1,2-propanediol, which PD and downstream enzymes are proposed to metabolize in other GRMs.^165^ Thus, GRM functions can be connected. Notably, not all functions of GRM-containing enzymes have been experimentally validated. Some functions have been predicted based on the annotations of GREs within the BMC loci, but as GRE eliminases share high sequence similarity, annotations can be imprecise. In other words, there may be additional functional variability within these GRM types and subtypes than is currently known.

Although all GRMs identified to date are specifically linked to GRE eliminases, it remains possible that future studies will uncover microcompartments associated with other GRE classes. One potential explanation for the apparent enrichment of GRE-associated microcompartments in secondary metabolic pathways is that these pathways are generally less branched and involve less substrate and/or intermediate sharing compared with primary metabolic pathways such as pyruvate metabolism, thus reducing the selective advantage of compartmentalization. As new microbial genomic data sets are sequenced, we expect other GRM classes will likely be discovered and functionally characterized.

Experimental studies of GRMs have remained limited despite the bioinformatic data that suggest that these GRMs are an abundant form of BMCs. Thus far, genetics and transmission electron microscopy studies have verified that GRM2s form and metabolize choline in *E. coli* 536 anaerobically.^166^ Interestingly, in this strain, the GRM2 locus is located in pathogenicity island II (PAI-II_536_), suggesting that this metabolic microcompartment might play a role in pathogenesis.^166^ Similarly, genetic studies identified that the GRM3 locus in *E. coli* CFT073 encodes for the 1,2-propanediol metabolizing microcompartment that is formed under anaerobic conditions.^167^ Genes essential for GRM3 formation and metabolic function were identified.^167^ Furthermore, propanediol-metabolizing activity of the GRM3 associated GRE, GRE-AE, and downstream aldehyde dehydrogenase from *Rhodopseudomonas palustris* BisB18 was confirmed through recombinant protein expression.^168^ Lastly, microscopy, proteomics, and enzymatic assays led to the determination that GRMguf is associated with IslA and isethionate metabolism in *Bilophila wadsworthia*, with these GRMs forming upon taurine addition anaerobically.^164^

Many questions regarding GRMs await investigation. One question is how GRMs function despite being “redox-replete bacterial organelles”, as described by Ferlez et al. in 2019.^169^ GRMs encapsulate GRE-AEs that require an electron source to sustain catalytic activity, yet they are physically sequestered in the BMC lumen away from cytoplasmic reductases. Current hypotheses for how this challenge is addressed include that shell proteins might shuttle electrons from the cytoplasm via metallocofactors, such as [4Fe-4S] clusters, or that GRMs might encapsulate additional redox proteins that generate reductants within the microcompartment.^169^ In 2014, Thompson et al. characterized a hexameric shell protein GrpU, named after its association with a glycyl-radical propanediol (Grp) microcompartment, that binds a [4Fe-4S] cluster within its central pore. This shell protein has been hypothesized to play a role in electron transport.^170^ Bioinformatic studies suggest that approximately 2/3 of all GRM loci encode a putative Fe-S cluster-binding shell protein.^169^ Similar shell proteins can be found in other BMC types that also encapsulate redox-active reactions. For example, comparative genomic analyses of BMC operons indicate that GrpU homologues are associated with B_12_-dependent, propanediol-utilizing (Pdu) BMCs, which contain a conserved GXCPQ motif that is responsible for Fe-S cluster binding.^170^ Additionally, a trimeric shell protein, with pseudohexagonal symmetry, named PduT is found in B_12_-dependent Pdu operons and is also proposed to bind a [4Fe-4S cluster].^171^ Experiments addressing the question of whether shell proteins bind electron-shuttling Fe-S clusters will provide insight into GRMs, and other BMCs that encapsulate redox-active proteins.

Other open questions include: why are GRE eliminases so commonly encapsulated within microcompartments, and what advantages are gained by GRM association? A primary proposed benefit for GRM formation is that compartmentalization would increase catalytic efficiency by controlling local concentrations of metabolites and enzymes and bringing them within close proximity to one another.^172,173^ Additionally, as aldehydes are toxic and volatile species,^174^ several studies have supported that compartmentalization protects the cell from toxic aldehyde intermediates produced by GREs (Figure 13).^175–179^ Lastly, an ongoing hypothesis in the field is that GRMs might protect the highly oxygen-sensitive GREs from oxygen damage.^163^ A similar function has been proposed for the prototypical BMC, carboxysomes, which are involved with carbon fixation; the carboxysome shell is thought to provide a diffusion barrier for the substrate CO_2_, and presumably O_2_.^180^ Successful efforts to engineer the oxygen-sensitive enzyme [FeFe]-hydrogenase into a synthetic, empty BMC shell resulted in a significant increase of aerobic catalytic activity in *E. coli*,^181^ thus strengthening the proposal that BMCs could confer oxygen protection. Whether or not GRMs result in oxygen protection, however, along with other putative advantages, remains an intriguing question awaiting experimental investigation.

Understanding the structure and function of GRMs could have important future applications. As GRMs are prominent in disease-associated anaerobes and studies suggest they might play a role in virulence,^182^ elucidating the role and structure of GRMs may lead to the identification of novel therapeutic targets for antibiotics. Additionally, if GRMs indeed confer advantages such as increased catalytic efficiency of GREs or protection against oxygen, engineering GREs to be encapsulated within synthetic microcompartments could improve the performance of these enzymes in environmental and industrial applications. Thus far, one study successfully demonstrated that PFL can be encapsulated within a synthetic microcompartment and is catalytically active,^173^ thus serving as a proof-of-concept foundation that engineering microcompartments for GREs could be a promising strategy for future applications.

## 7 Summary and Outlook

The glycyl radical enzyme (GRE) superfamily, initially founded with just three distinct members, now spans a wide range of transformations. Driven by the explosion of metagenomic data and improved methods for expressing, handling, and assaying these oxygen-sensitive enzymes, the number of functionally and structurally characterized GREs continues to grow. The unifying feature of this superfamily is the use of amino acid-based radicals as cofactors. In all characterized systems to date, catalysis relies on a division of labor between a persistent “radical reservoir” and a transient “reactive radical”.^99,183^ The reservoir is a Cα-centered radical on a proteinogenic amino acid (classically glycine) that enables formation of a cysteine-derived thiyl radical, which serves as the catalytic workhorse, typically by hydrogen atom transfer. This design is economical because the radical reservoir needs to be installed only once.^7^ After that, tens of thousands of turnovers can proceed without further input of cofactors or reductant.^53^

As the GRE superfamily has not been comprehensively reviewed for almost a decade,^4,99,184,185^ we reassessed how GREs are classified. The initial division between “RNR-like” and “PFL-like” members still holds, and our updated framework applies only to the “PFL-like” GREs. Since the last comprehensive review,^99^ many new eliminase functions have been characterized; accordingly, we subclassified these enzymes by the specific bond-cleavage chemistry they catalyze. In addition, a distinct class of GRE isomerase emerged with the discovery of three such enzymes in the early 2020s.^39,87^ Two of these isomerases enable an ‘anhydroglycolysis’ pathway in common gut bacteria, revealing an overlooked route for carbohydrate metabolism and raising the potential biological relevance of 1,5-anhydroglucitol and 1,5-anhydromannitol.^39^ The superfamily has also expanded in architectural complexity. Though accessory subunits have long been known for select GREs (HPAD and BSS),^70,71^ MASS was recently shown to include a rubredoxin-like δ subunit that binds a single Fe ion.^77^ This feature has not yet been observed in GREs outside of MASS, and the role of this subunit remains to be defined. Finally, a paradigm shift in 2024 established that the radical reservoir need not be restricted to glycine; alanine, serine, and threonine can also serve as radical-storage residues.^47^ No functions for these AAREs have yet been characterized, and they represent an exciting opportunity to uncover new biology and chemistry within the superfamily.

Many of the newly discovered GRE decarboxylases, eliminases, and isomerases are encoded by anaerobes within the human microbiome (e.g., gut, oral, and genital), linking GRE chemistry to host-relevant metabolism. Hydroxyproline utilization pathways tap into collagen-derived substrates, and GREs that enable this metabolism have been proposed as potential antimicrobial targets because they lack a human homologue.^37,105^ GRE-mediated C–N bond cleavage has been associated with trimethylamine production (and downstream trimethylamine N-oxide), which has been linked to cardiovascular disease, and with metabolic disease contexts such as nonalcoholic fatty liver disease.^90^ GRE-mediated C–S bond cleavage can generate sulfite and ultimately hydrogen sulfide, a biologically active metabolite with disease associations.^157^ Several well-studied GRE decarboxylases also produce malodorant, host-active small molecules: HPAD and AAD generate the uremic toxin *p*-cresol, PAD produces toluene, and IAD produces skatole.^129–131^ Skatole, in particular, has long been recognized as both malodorant and toxic, with implications spanning human health and disease as well as environmental and agricultural contexts.^132^ Continued study of GREs prevalent in mammalian microbiomes will sharpen our understanding of host–microbe chemical exchange at the molecular level and may reveal new opportunities to modulate these pathways for therapeutic benefit.

Although functional and structural annotation has accelerated in the past decade, many GREs remain unannotated.^105^ Motif searches and sequence-clustering approaches (e.g., sequence similarity networks) can help predict function and prioritize targets, yet entire clades still lack experimental validation. This gap is especially clear for archaeal GREs. All PFL-like GREs with characterized functions to date are bacterial, but archaeal homologues are also known to exist. One such archaeal PFL-like GRE has been structurally characterized, but its function has been debated for nearly two decades.^95,186^ It was initially proposed to be a GD,^95^ whereas more recent work has suggested it may instead be a MASS.^186^ Either assignment would be of practical interest given its hyperthermophilic origin, since a GD could convert biodiesel-derived glycerol into propane-1,3-diol,^187,188^ and MASS chemistry has relevance to organic synthesis, bioremediation, and energy-related applications.^189,190^ This archaeal case, along with many other unannotated clades, illustrates the broader challenge of metagenomics outpacing biochemistry.

Computational tools can generate strong hypotheses, but functional characterization ultimately requires experiments. For many GREs, a major bottleneck is reconstituting the active, glycyl-radical form of the enzyme. In particular, the radical SAM activating enzymes required to install the glycyl radical are often insoluble or low-yielding when produced in *E. coli*. Even when soluble protein is obtained, reconstitution of the catalytic [4Fe–4S] cluster can be challenging. New, out-of-the-box strategies for generating the glycyl radical in vitro could therefore accelerate GRE discovery, functional assignment, and downstream applications. In parallel, increasingly sophisticated protein prediction^191^ and design tools^192^ may help address these limitations by enabling more stable GRE:AE interfaces, improved solubility in *E. coli*, and greater AE stability.

The XSSs (GRE hydroalkylases) are a prime example of how activation challenges have limited both mechanistic and application-driven studies. By identifying homologous activating enzymes that are soluble and, in some cases, cross-reactive,^32,109^ we can begin to overcome this bottleneck and unlock broader GRE hydroalkylase potential. From a synthetic perspective, GRE hydroalkylases offer a direct route from simple, achiral substrates to stereochemically complex, sp^3^-rich products, with clear appeal for drug discovery and scalable synthesis. Hydroalkylation provides an efficient approach to C­(sp^3^)–C­(sp^3^) bond formation, establishing up to three contiguous stereocenters, including up to two quaternary centers, in a single step with high atom economy (no prefunctionalization and minimal byproduct formation).^193^ More broadly, radical enzymes can achieve a level of precision in C–H activation that remains difficult for small-molecule catalysis to match, underscoring the untapped potential of radical biocatalysis. With robust activation now in hand,^32,194^ the field can more systematically evaluate substrate scope, stereocontrol on non-native substrates, and tunability. If these properties prove general, the next step will be to develop high-throughput activation and hydroalkylation workflows to engineer improved specificity, broaden substrate scope, and ultimately demonstrate scalability.

GREs represent a radical platform that Nature has repeatedly repurposed to achieve diverse anaerobic transformations. Recent discoveries, including GRE isomerases^39,87^ and aminoacyl radical enzymes (AAREs),^47^ along with emerging multisubunit assemblies, are already reshaping long-held assumptions about what this superfamily can do and how it is built. Looking ahead, progress will hinge on overcoming practical barriers to activation and on capturing catalytically relevant structural states. Meeting these challenges should accelerate functional assignment across the rapidly expanding sequence space, and practical leverage to engineer GREs as synthetic biocatalysts and to target GRE-mediated chemistry implicated in human health.

## Abbreviations

5′dAdo•: 5′-deoxyadenosyl radical
AAD: arylacetate decarboxylase
AARE: aminoacyl radical enzyme
AdoMet: *S*-adenosyl-L-methionine
AG6P: 1,5-anhydroglucitol-6-phosphate
AM6P: 1,5-anyhydromannitol-6-phosphate
BMC: bacterial microcompartment
BSS: benzylsuccinate synthase
*c*4L-NMHP: N-methyl-*cis*-4-hydroxy-L-proline
*c*4D-NMHP: N-methyl-*cis*-4-hydroxy-D-proline
CHT: choline
CoA: coenzyme A
CSS: complex significance score
Cryo-EM: cryogenic electron microscopy
CutC: choline trimethylamine lyase
DF6P: 1-deoxyfructose-6-phosphate
DHPS: 2-dihydroxypropanesulfonate
EPR: electron paramagnetic resonance spectroscopy
FMO: flavin-containing monooxygenase
FUM: fumarate
GD: glycerol dehydratase
GOL: glycerol
GRD: glycyl radical domain
GRE: glycyl radical enzyme
GRE-AE: glycyl radical enzyme activating enzyme
GRM: glycyl radical enzyme microcompartment
HAT: hydrogen atom transfer
HBSS: 4-hydroxybenzylsuccinate synthase
HiPIP: high potential iron-sulfur protein
HPA: 4-hydroxyphenylacetate
HPAD: 4-hydroxyphenylacetate decarboxylase
HpfG: dihydroxypropanesulfonate dehydratase
HplG: *trans*-4-hydroxy-D-proline C–N lyase
HpsG: 2-hydroxypropanesulfonate sulfolyase
HpyG: N-methyl-*cis*-4-hydroxy-L-proline C–O lyase
HpzG: N-methyl-*cis*-4-hydroxy-D-proline C–O lyase
HypD: hydroxyproline dehydratase
I3A: indole-3-acetate
IAD: indoleacetate decarboxylase
IBSS: 4-isopropylbenzylsuccinate synthase
Isl: isethionate
IslA: isethionate sulfite lyase
IseG: isethionate sulfite lyase
LSS: limonenylsuccinate synthase
MASS: 1-methylalkylsuccinate synthase
MBN: toluene
NMSS: naphthyl-2-methylsuccinate synthase
PflD: 1,5-anyhydromannitol-6-phosphate C–O lyase
PA: phenylacetate
PAD: phenylacetate decarboxylase
PD: propanediol dehydratase
PDO: propane-1,2-diol
PFL-AE: pyruvate formate lyase activating enzyme
PFL: pyruvate formate lyase
PYR: pyruvate
RNR: ribonucleotide reductase
RS: radical *S*-adenosyl-L-methionine
SEC: size exclusion chromatography
*t*4D-HP: *trans*-4-hydroxy-D-proline
*t*4L-HP: *trans*-4-hydroxy-L-proline
TdcE: 2-ketobutyrate formate lyase
TMA: trimethylamine
TMAO: trimethylamine oxide
XSS: X-succinate synthase
YbiW: 1,5-anhydroglucitol-6-phosphate C–O lyase
YfiD: autonomous glycyl radical cofactor
